# Curdione combined with borneol treats bacterial mixed HPV infection by regulating the crosstalk among immune cells

**DOI:** 10.3389/fimmu.2025.1503355

**Published:** 2025-01-22

**Authors:** Jingwei Liu, Tong Shu, Yiheng Mu, Wanlin Zheng, Xiaohuan Lu, Hong Tao

**Affiliations:** ^1^ Department of Gynecology, Wuhu Maternal and Child Health (MCH) Center, Wuhu, China; ^2^ School of Clinical Medicine, Wannan Medical College, Wuhu, China; ^3^ Graduate School, Wannan Medical College, Wuhu, China; ^4^ Department of Plastic Surgery, The Second Affiliated Hospital of Jiangxi Medical College, Nanchang University, Nanchang, China; ^5^ Jiangxi Province Key Laboratory of Immunology and Inflammation, Jiangxi Provincial Clinical Research Center for Laboratory Medicine, The Second Affiliated Hospital of Jiangxi Medical College, Nanchang University, Nanchang, China

**Keywords:** human papillomavirus, bacterial vaginosis, immunocyte crosstalk, curdione, borneol, network pharmacology

## Abstract

**Background:**

Human papillomavirus (HPV) infection is a worldwide reproductive system disease. Baofukang suppository, a traditional herbal preparation that includes curdione and borneol, has been reported to treat bacterial vaginosis (BV) and HPV infection in China. However, the therapeutic mechanism is still unknown. This study aims to explore the molecular mechanisms of curdione and borneol in treating HPV infection.

**Methods:**

We conducted a retrospective cohort analysis of medical records from a single-center study involving 205 HPV patients, focusing on the correlation between HPV clearance and co-infection with other pathogens, confirming the efficacy of Baofukang suppository. Bioinformatics and network pharmacology approaches were employed to identify therapeutic targets of Baofukang suppository for BV/HPV co-infections. qRT-PCR, Western blot, immunofluorescence staining, and flow cytometry were utilized to validate the therapeutic targets of curdione and borneol, along with the associated immune molecular changes. Finally, the molecular mechanisms and therapeutic efficacy of curdione and borneol were confirmed *in vivo* using an LPS/TC-1 cervical orthotopic injection model.

**Results:**

Curdione and borneol selectively inhibit the secretion of interleukin-6 (IL-6) and interleukin-1β (IL-1β) by macrophages. The reduction in IL-6 and IL-1β levels effectively inhibits the expression of CD274 (Programmed death ligand 1, PD-L1) in infected epithelial cells by inhibiting STAT3 phosphorylation, thereby suppressing their immune evasion capabilities. Furthermore, curdione and borneol enhance the expression of tumor necrosis factor α (TNF-α) and caspase 1 (CASP1) in macrophages, as well as the expression of interleukin 12 (IL-12) and interleukin 23 (IL-23) in dendritic cells (DCs). The expression of these inflammatory factors effectively promotes the migration and differentiation of T cells to the site of infection, completing the clearance of infected epithelial cells.

**Conclusion:**

The main components of Baofukang suppository, curdione and borneol, inhibit the progression of HPV infection and the occurrence of cervical cancer by modulating the communication between innate and adaptive immunity, promoting the recruitment and recognition of CD8^+^ T cells to eliminate HPV-infected epithelial cells.

## 1 Introduction

HPV infection is one of the most common sexually transmitted infections (STIs) in the world, and it primarily causes genital warts and cervical cancer (1). Although most female patients can clear HPV infection through spontaneous immunity within 6-18 months, the occurrence of mixed infections with other pathogens can lead to persistent HPV infection (2–4). The correlation between BV and HPV infection has been reported in several clinical cohort studies worldwide, and BV infection disrupts the balance of the vaginal flora thereby enhancing HPV susceptibility (3, 5–7). However, current research suggests that beyond the vaginal microbial environment, both the vaginal epithelial barrier and the mucosal immune response play pivotal roles in HPV infection (8–10).

Innate and adaptive immune responses, constituting the primary defenses against pathogens residing on the vaginal mucosal surface, are indispensable for the elimination of HPV infection (10, 11). Upon activation by pathogen-associated molecular patterns, innate immune cells secrete diverse cytokines to control the progression of HPV infection (8, 12). Adaptive immunity activates cytotoxic T-lymphocytes (CTLs) in response to antigen presentation by antigen-presenting cells (APCs) and cytokine signaling to eliminate HPV-infected epithelial cells (13). However, in contrast to other viruses, HPV does not induce epithelial cell lysis, resulting in a significant reduction in antigen presentation by macrophages and DCs (14). Furthermore, HPV has been reported to inhibit DC maturation and downregulate the expression of major histocompatibility complex (MHC) molecules through mechanisms involving interleukin-10 (IL-10), transforming growth factor-β (TGF-β), arginase 1 (Arg-1), or indoleamine 2, 3-dioxygenase (IDO-1), thereby preventing the clearance of infected cells by CTLs (15–18).

It is puzzling that while HPV achieves immune evasion by inhibiting APC cells, an overly and persistently activated immune system in co-infection with BV fails to effectively eliminate HPV (19–21). In hepatoma cells, CD274 expression is stimulated by high levels of inflammatory cytokines IL-6 and IL-1β via the STAT3 signaling pathway to enable immune evasion (22). Elevated CD274 expression and impaired T-cell function have also been reported in HPV-associated head and neck squamous cell carcinomas (HNSCCs) and cervical cancer (23–25). Similarly, increased CD274 expression is found in HPV-infected epithelial cells (26). These studies suggest that in the bacterial mixed HPV infection, the persistent HPV infection is not solely due to the damage of vaginal epithelial barrier and microbial environment, but rather involves more complex immune evasion mechanisms.

Baofukang suppository, as a traditional Chinese medicine preparation, is widely used in the clinical treatment of BV in China. In recent years, several researchers have reported that it has also demonstrated satisfactory therapeutic effects in HPV infection (27–29). Baofukang suppositories consist of natural borneol and zedoary oil, which are extracted from the branches and leaves of Cinnamomum camphora (Linn.) Presl and the rhizomes of Curcuma zedoaria (Christm.) Rosc, respectively (30). Borneol and Dl-isoborneol, the primary active components of natural borneol, exhibit diverse biological activities, including anti-inflammatory, antioxidant, anti-apoptotic, and anticoagulant properties (31). Previous literature has reported that borneol ameliorates neuroinflammation in post-stroke mice by modulating immune cell polarization (32). The major active constituents of zedoary oil are terpenes, such as epicurzerenone, curdione, and 1,8-cineole, which are being researched in cancer treatment (30, 33). Curdione has been reported to influence tumor immunity and promote tumor cell apoptosis through IDO1 (34). Although the bioactivities of the primary active components in Baofukang suppository are significant, the molecular mechanisms underlying their therapeutic effects in HPV and BV remain unknown. Furthermore, whether they exhibit synergistic effects in immune regulation warrants further investigation.

In this study, we found that Baofukang suppositories exhibit promising therapeutic effects against bacterial-mixed HPV infections. Employing systems pharmacology, we identified the key active ingredients, curdione and borneol, along with their specific targets of action. Furthermore, we validated that curdione and borneol facilitate HPV clearance by modulating the interplay between innate and adaptive immune cells. Curdione and borneol blocked the IL-6/IL-1β-STAT3-CD274 axis-mediated HPV immune escape by inhibiting excessive immune activation. Meanwhile, they promoted the secretion of adaptive immune-related cytokines by macrophages and DCs, accelerating the maturation and focal infiltration of CD3^+^ CD8^+^ T cells. This study proposes an HPV immune escape mechanism under BV/HPV co-infections and explains that curdione combined with borneol achieves HPV clearance through the crosstalk among immune cells. This study aims to establish a theoretical basis for the formal adoption of Baofukang suppositories as an efficacious therapeutic modality for HPV infection.

## 2 Materials and methods

### 2.1 Reagents

Curdione (C418576) and borneol (B119291) were purchased from Aladdin (China). Baofukang suppositories (Z46020058) were purchased from Bikai Pharmaceutical (China). LPS (L2880) was purchased from Sigma-Aldrich (USA). CpG (GC68897), and HPV E6 protein (GP25441) were purchased from GLPBIO (USA).

Antibodies: iNOS (22226-1-AP), TLR4 (66350-1-Ig), NF-κB p65 (10745-1-AP), CD274 (66248-1-Ig), ERK1/2 (66192-1-Ig), AKT (60203-2-IG) and STAT3 (60199-1-IG) were purchased from Proteintech (China). Antibodies: IL-6 (DF6087), p-STAT3 (AF3293), p-ERK1/2 (AF1014) and p-AKT (AF0016) were purchased from Affinity (USA). TLR9 (13674) and CD8a (98941) antibodies were purchased from CST (USA). Flow cytometry antibody: FITC-CD3 (E-AB-F1013C), APC-CD8a (E-AB-F1104E), PE-CD8a (E-AB-F1104D), APC-CD4 (E-AB-F1097E) and PE-CD4 (E-AB-F1097D) were purchased from Elabscience (China).

### 2.2 Retrospective cohort analysis

A retrospective cohort analysis was conducted to assess the therapeutic efficacy of Baofukang suppository in patients with bacterial mixed HPV infection. With the informed consent of the participants and ensuring their privacy, we collected the medical information and test results of female patients with HPV vaginal infection who visited the Gynecological Clinic of Wuhu Maternity and Child Healthcare Hospital in Anhui Province from June 2022 to June 2023. Patients were grouped based on their medical information, and underwent follow-up visits, with reassessments of vaginosis and HPV infection status at 3 and 5 months after the consultation. Two authors independently assessed patients for inclusion and risk of bias, extracted data and checked them for accuracy. All patients received basic treatment according to the 2021 Sexually Transmitted Infections Treatment Guidelines and were subsequently administered Baofukang suppositories in combination therapy based on their individual preferences (21). As a traditional Chinese medicine preparation, Baofukang suppository, while clinically proven beneficial for patients with HPV or BV infections, is not considered a necessary treatment option. Some patients are unwilling to undergo the treatment due to its vaginal administration route.

Excluding invalid data, a comprehensive dataset of 205 cases was successfully amassed for analysis. The valid data were categorized into two groups: a control group (n = 104) and a Baofukang suppository-treated group (n = 101), based on the administration status of Baofukang suppositories. This clinical cohort study revealed no statistically significant differences between the two groups of participants in terms of baseline characteristics and vaginal pathogenic microorganism examination indicators, including mean age, pregnancy history, delivery history, age at first pregnancy, sexual intercourse frequency, microbial diversity, microbial density, Nugent score, and Amsel (AV) criteria level (**Supplementary Table 1**, *P* > 0.05). The study protocol was approved by the Ethics Committee of Wuhu Maternal and Child Health Center (**Approval Number: 20230006**).

### 2.3 Patient information inclusion and exclusion criteria

Inclusion criteria: 1. Female individuals with a history of sexual intercourse; 2. Meeting the relevant diagnostic criteria for infection with high-risk HPV types, with positive results in high-risk HPV tests; 3. No sexual intercourse, vaginal lavage, or intravaginal medication use within 3 days before the examination; 4. No receipt of any anti-HPV treatment within 4 weeks before consultation; 5. Patients with high-risk HPV positivity, undergoing colposcopy and biopsy, with pathological findings indicating inflammation or low-grade lesions; 6. Fulfilling the medication indications of this study; 7. Having signed the informed consent form.

Exclusion Criteria: 1. Presence of malignant tumors or precancerous lesions confirmed by pathological examination; 2. Existence of immune system or hematological disorders; 3. Concurrent dysfunction of other vital organs; 4. Cognitive or communication impairments; 5. Failure to adhere to medication instructions or discontinuation of treatment; 6. Pregnancy or lactation; 7. Receipt of other traditional Chinese medicine treatments within 7 days before the intervention; 8. Loss to follow-up.

### 2.4 Medication protocol for Baofukang suppository

Patients should administer one Baofukang suppository into the posterior fornix of the vagina after cleansing the external genitalia before bedtime, during non-menstrual periods. The administration should be done every other day, with a total of 10 administrations comprising one treatment course. Continuous treatment for three courses is recommended. During the treatment period, patients are advised to avoid bathtub baths and sexual intercourse, refrain from overwork, and ensure proper scheduling of rest and moderate exercise to enhance their immune resistance.

### 2.5 BV and HPV data collection

The RNAseq data for vaginal swabs of BV infection were sourced from the GSE113771 project in the GEO database (https://www.ncbi.nlm.nih.gov/geo/), where samples from BV-related (n = 3) and healthy (n = 3) subjects were collected (22). The RNAseq data for vaginal swabs of HPV infection were sourced from the GSE75132 project in the GEO database, where samples from high-risk HPV-infected individuals without cervical cancer (n = 10) and healthy (n = 11) subjects were collected (23). The normalization of sequencing data was accomplished via GEO2R (https://www.ncbi.nlm.nih.gov/geo/geo2r/).

### 2.6 Collection of active ingredients and targets from natural borneol and zedoary oil

The main active compounds of natural borneol and zedoary oil were collected from related literature, utilizing databases such as the Traditional Chinese Medicine Network pharmacology database (24) (TCMSP, https://old.tcmsp-e.com/tcmsp.php), the Traditional Chinese Medicine Integrated Database (25) (TCMID, http://www.megabionet.org/tcmid/), the Herbal Ingredients Targets Database (26) (HIT, http://hit2.badd-cao.net/), and other relevant sources (20). The detailed molecular information is presented in **Supplementary Table 2**. Given that Baofukang suppository is intended for intravaginal use, the oral bioavailability of its main active ingredients is not a determining factor. Subsequently, drug target prediction for the major active ingredients was conducted using Swiss target prediction (**Supplementary Table 3**).

### 2.7 Collection of disease targets for BV and HPV vaginal infections

The target genes associated with BV and HPV vaginal infections were collected and organized from GeneCards database (27) (https://www.genecards.org/) and DisGeNET (28) (http://www.disgenet.org/). Detailed disease targets are presented in **Supplementary Excel 1**.

### 2.8 Univariate and multifactorial Cox analysis

To evaluate the correlation between patients’ baseline characteristics (including mean age, pregnancy history, delivery history, age at first pregnancy, and sexual intercourse frequency) and indicators related to vaginosis (including microbial diversity, microbial density, Nugent level, AV level, and bacterial infection type) with HPV clearance rate, a Cox analysis is conducted. The univariate and multivariate Cox analysis was conducted by the survival coxph function of the R package.

### 2.9 Enrichment analysis of GO and KEGG pathway

The Gene Ontology database (GO, https://www.geneontology.org) groups terms of gene and gene product function into three different dimensions of the ontology, Cellular Component (CC), Molecular Function (MF), and Biological Process (BP), to help researchers identify sets of genes that are significantly enriched in specific biological processes. The Kyoto Encyclopedia of Genes and Genomes (KEGG, https://www.genome.jp/kegg) is a database for systematic analysis of gene function to identify the relevance of candidate targets in the corresponding biological functions and signaling pathways. GO and KEGG pathway analysis was performed by linking targets to the Database for Annotation, Visualization and Integrated Discovery (DAVID, https://david.ncifcrf.gov) (29). The top 20 GO terms and KEGG pathways with the smallest p-values were selected and uploaded to the bioinformatics mapping website (https://www.bioinformatics.com.cn) for graph generation.

### 2.10 PPI network and TISSUS enrichment analysis

The selected gene sets were imported into the STRING database (https://cn.string-db.org/) for PPI network analysis and TISSUS enrichment analysis, with confidence scores set to ≥ 0.7 (30, 31). Cytoscape 3.7.1 was used for data visualization for PPI network analysis (32). The CytoHubba plug-in within Cytoscape was employed to identify hub genes and rank them based on the MCC score (33).

### 2.11 Lasso regression analysis

Least Absolute Shrinkage and Selection Operator (LASSO) is a regression analysis method that performs gene selection and classification. Separate LASSO regression analyses were performed on the set of disease targets for BV and HPV vaginal infections to identify signature genes, using the R package “glmnet” (34). Characterization genes are detailed in **Supplementary Table 4**.

### 2.12 CIBERSORT immune infiltration analysis

CIBERSORT can use deconvolution with a collection of 22 immune cell signature genes to derive the proportion of various immune cells from tissue RNAseq data (35). For identifying potential immune cell targets related to BV and HPV vaginal infections, immune infiltration analyses were performed on co-DEGs, utilizing the R package “CIBERSORT”.

### 2.13 Cell culture and treatment

Lung epithelial cells (TC-1) expressing mouse HPV E6/E7 proteins, macrophages (Raw 264.7) and DCs (DC 2.4) were cultured in RPMI 1640 medium (Gibco, USA) supplemented with 10% FBS (Gibco, USA) in a humidified incubator containing 5% CO2. The co-culture model was based on Transwell 12 well plates (3401, Corning, USA), where DCs were mixed with macrophages in a 1:3 ratio in the upper chamber, and TC-1 cells were cultured in the lower chamber and synchronized with splenic lymphocytes in the stimulated addition chamber. Splenic lymphocytes were added to the lower chamber at the time of administration of the stimulus. Under unspecified conditions, Baofukang suppository, curdione and Borneol were all stimulated at 100 μg/mL for 24 hours. LPS stimulation at 2 μg/mL was employed to establish a model of bacterial infection-associated inflammation. To mimic a viral infection-associated inflammatory response, cells were stimulated with 1 μg/mL CpG in combination with 10 μg/mL HPV E6 protein. In the co-culture model, 20 ng/mL of IL-6 and IL-1β were used to induce CD274 expression in TC-1 cells.

### 2.14 Quantitative real-time PCR

For the qRT-PCR assay, total RNA was extracted from treated cells using Trizol reagent (Invitrogen, USA). 3 μg of mRNA was reverse transcribed to cDNA and qRT-PCR was performed to detect mRNA expression of specific genes (Vazyme, China). The primer sequences are shown in **Supplementary Table 5**.

### 2.15 Western blot analysis

For the WB assays, cells were treated with RAPI lysate (Biosharp, China) containing protease and phosphatase inhibitors. The protein concentration was determined by the BCA Protein Assay Reagent (Pierce, USA). 45 μg of protein from each sample was separated by electrophoresis (10%, w/v) and transferred to the NC membranes (Amersham, USA). Membranes were blocked with 5% BSA (Biosharp, China) for 1 hour and then incubated with the corresponding antibodies overnight. After washing with TBST, they were incubated with secondary antibodies (Proteintech, USA) for 2 hours. Following another wash, the membrane was visualized by Universal Hood II (BIO-RAD, USA). The gray value of the bands was quantified using ImageJ.

### 2.16 Immunofluorescence staining

Immunofluorescence staining was performed to detect the expression level and spatial distribution of specific proteins. The treated cells were fixed with 4% paraformaldehyde for 15 minutes at room temperature. After permeabilization with 0.1% Triton X-100 for 10 minutes, cells were blocked with 3% BSA for 30 minutes. After 3 washes with PBS, the cells were incubated overnight at 4°C with the indicated primary antibody and then with the fluorescent secondary antibody (Proteintech, USA) for 1 hour at room temperature.

### 2.17 Antibacterial experiment

Gardnerella vaginalis (ATCC 14018) was obtained from the American Type Culture Collection (ATCC). Colonies were inoculated onto blood agar plates and cultured in a 5% CO_2_ atmosphere. Gradient dilutions were performed to ensure that Gardnerella vaginalis grew as individual colonies in the antimicrobial assay. Bacterial solutions were mixed with 100 μg/mL of Baofukang suppositories or Curdione + borneol, respectively, and then inoculated. The colonies were counted after 24-48 hours of incubation.

### 2.18 Experimental animals and model construction

Female C57BL/6 mice (aged 6-8 weeks, weighing 18-20 g) were purchased from the Liaoning Changsheng Laboratory Animal Center (China) and maintained in an SPF-grade environment. Mice were anesthetized by intraperitoneal injection of 0.4ml/20g of 0.3% pentobarbital sodium. BV/HPV co-infections were mimicked by injecting 20 μL of TC-1 cell suspension (5 × 10^5^ cells/mL) mixed with 5 μg of LPS into the cervical wall tissue of mice. After the LPS/TC-1 cervical *in situ* injection model had been established for 4 days, vaginal lavage treatment was administered according to the group assignments. The vaginal lavage treatment consisted of a single dose of 0.5 mL, administered once every two days, for a total of seven treatments. Animal procedures and care were guided by the Animal Research: Reporting *In Vivo* Experiments (ARRIVE) guidelines. All protocols related to animal experiments in this study were approved by the Ethics Committee of Wuhu Maternal and Child Health Center (**Approval Number: 2024009**).

### 2.19 Extraction of mouse vaginal mesenchymal stromal cells and splenic lymphocytes

To extract mesenchymal cells from the vagina and cervix, the corresponding tissues were collected and minced. The tissues were digested with 0.125% trypsin and 0.8 mg/mL collagenase I at 37°C for 1 hour. After digestion, the tissues were passed through a 200-mesh strainer to obtain a single-cell suspension, which was subsequently cultured in RPMI 1640 medium containing 10% FBS for biocompatibility experiments.

For lymphocyte extraction, the appropriate tissues were collected and crushed in a mortar. The mixture was passed through a 200-mesh strainer. Cells were then collected by centrifugation, and excess erythrocytes were removed using an erythrocyte lysate solution. The single-cell suspensions were washed with PBS to obtain a pure single-cell suspension for flow cytometry.

### 2.20 Flow cytometry

The treated cells were collected and stained with appropriate concentrations of fluorescently labeled antibodies for 30 minutes, protected from light, at 37°C. After washing with PBS, the stained cells were stored in 2% paraformaldehyde awaiting detection. Flow cytometry was performed by LSRFortessa X-20 (BD, USA) and the data were analyzed by FlowJo.

### 2.21 H&E and immunohistochemical staining

For H&E staining, paraffin tissue sections were first deparaffinized, and then stained with hematoxylin for 5 minutes. After washing with ddH_2_O, they were stained with eosin for 1 minute. After transparentization with alcohol and xylene, neutral resin was used to seal the slides.

For immunohistochemical staining, antigen retrieval was performed in citrate buffer solution (0.01 M) after deparaffinization of paraffin tissue sections. After treatment with 3% H_2_O_2_, the sections were incubated with the indicated antibodies overnight at 4°C. The following day, the sections were washed with PBS and incubated with the secondary antibodies for 30 minutes at room temperature. Subsequently, 3,3’-Diaminobenzidine (DAB) and hematoxylin were used for staining.

### 2.22 Statistical analysis

One-way analysis of variance (ANOVA) was used to compare data between multiple groups, while Student’s t-test was used to compare data between two groups. GraphPad Prism 8.0.2 is utilized for graphing and statistical analysis. Non-quantitative data from the clinical cohort information were compared using the nonparametric Mann-Whitney U test by SPSS 25.0. The threshold for statistical significance was set at *P* < 0.05.

## 3 Results

### 3.1 Clinical efficacy of HPV with Baofukang suppository

We collected the medical information from patients diagnosed with vaginal HPV infection who visited a single center within one year from June 2022 and followed up continuously, with a total of 205 complete cases collected. Patients were categorized into control and Baofukang groups based on whether they were treated with Baofukang suppositories or not, and all patients with concurrent vaginosis were routinely treated. There were no group differences in age, sexual frequency, and number of pregnancies between the control and treatment groups (**Supplementary Table 1**).

**Figure 1:**
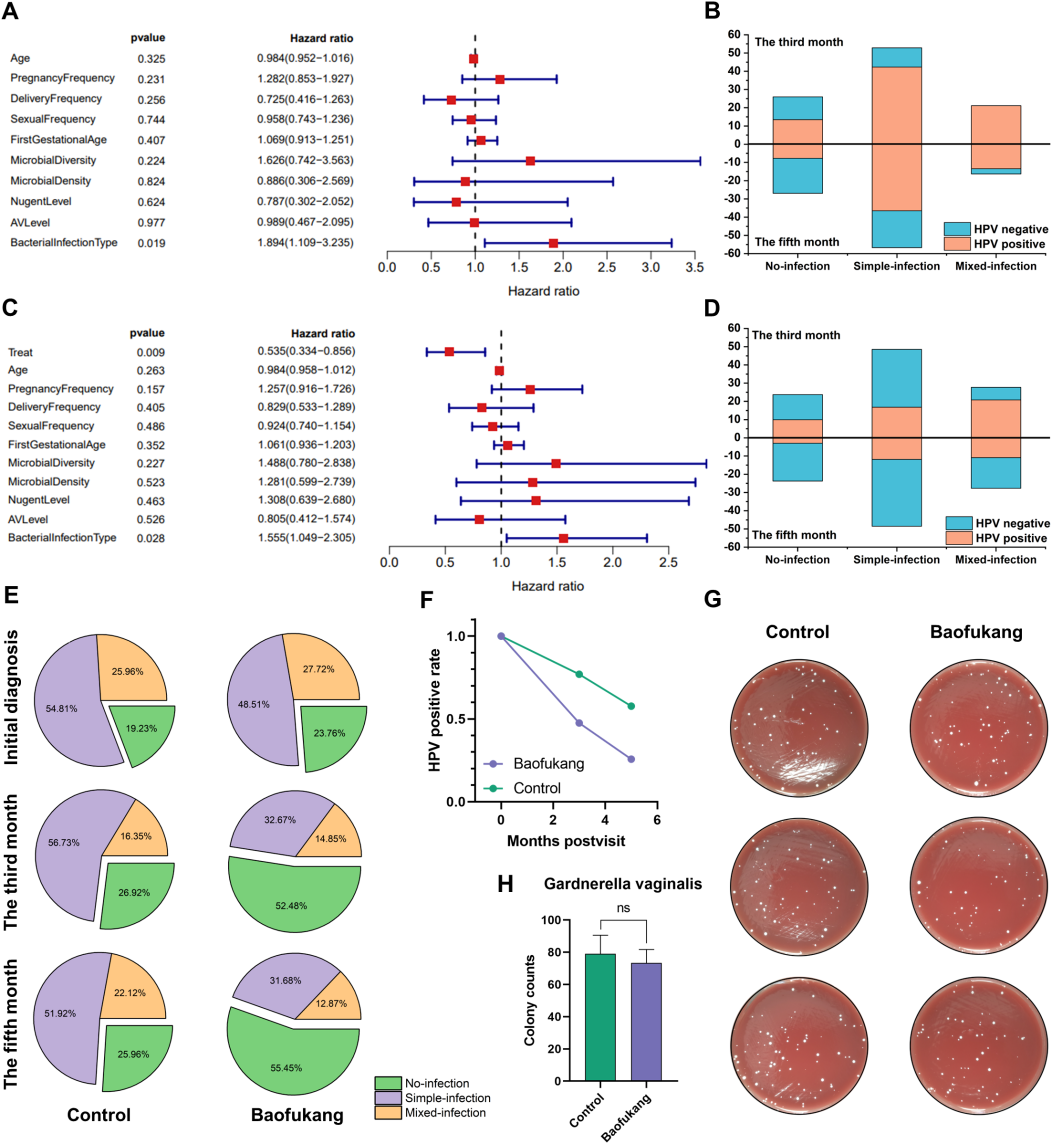
Baofukang suppositories facilitate HPV-negative conversion in cases of bacterial mixed HPV infection and restore the vaginal microbial environment. **(A)** Multifactorial Cox analysis of a cohort of 104 HPV patients who were not given pharmacological interventions for HPV treatment (control group); **(B)** Analysis of additional vaginal pathogen infections versus HPV-negative conversion in the control group at the third versus fifth month after consultation (No-infection: Someone infected with HPV only; Simple-infection: Someone infected with HPV and an additional pathogen; Mixed-infection: Someone infected with HPV and multiple additional pathogens.); **(C)** Multifactorial Cox analysis on a cohort consisting of control group and 101 HPV patients who were administered Baofukang suppositories (Baofukang group); **(D)** Statistical graph of additional vaginal pathogen infections versus HPV negative conversion in the control group versus the Baofukang group at the third versus fifth month after consultation; **(E)** The control and Baofukang groups with additional pathogen infections in the vagina at the time of consultation, and at the third and fifth month after consultation; **(F)** HPV- negative conversion in the control and Baofukang groups versus time; **(G)** Bactericidal properties of Baofukang suppositories against Gardnerella vaginalis at no-cytotoxic concentration (cytocompatibility, **Supplementary Figure 2**) and **(H)** the quantification of colonization (n = 3). Data are shown as mean ± SD; *P* values were calculated using Student’s t-test.

We investigated the impact of HPV-negative conversion in patients receiving conventional treatment. Multivariate Cox analysis showed only the type of initial bacterial infection was an independent influencing factor in the control group (**Figure 1A**). Although HPV will gradually be cured over time, the type of initial bacterial infection will affect the difficulty of HPV curing (**Figure 1B**). Introducing the Baofukan group and conducting a multivariate Cox analysis, the results showed that both the treatment with Baofukang suppository and the type of initial bacterial infection were independent influencing factors (**Figure 1C**). The complexity of initial bacterial infection still affected HPV cure in patients, but Baofukang suppository was effective in promoting cure of bacterial mixed HPV infections (**Figure 1D**). Concurrently, Baofukang suppository also mitigated the complexity of bacterial infection (**Figure 1E, F**). Nevertheless, we found that at therapeutic concentrations, Baofukang suppositories exhibited no significant bactericidal impact against Gardnerella vaginalis, a common pathogen associated with bacterial vaginosis (**Figure 1G, H**). Baofukang suppository can effectively promote the cure of HPV/BV co-infections, but not by directly killing the mixed infection pathogens and the mechanisms remain to be further investigated.

### 3.2 Shared disease characteristics of BV and HPV vaginal infections

Bacterial infections in this cohort of HPV patients were predominantly BV alone, or BV combined with aerobic vaginitis (AV) or vulvovaginal candidiasis (VVC) infections, with mixed BV infections being the most prevalent (106/161). Consequently, BV was selected as a representative case of additional pathogen infection for further investigation.

**Figure 2:**
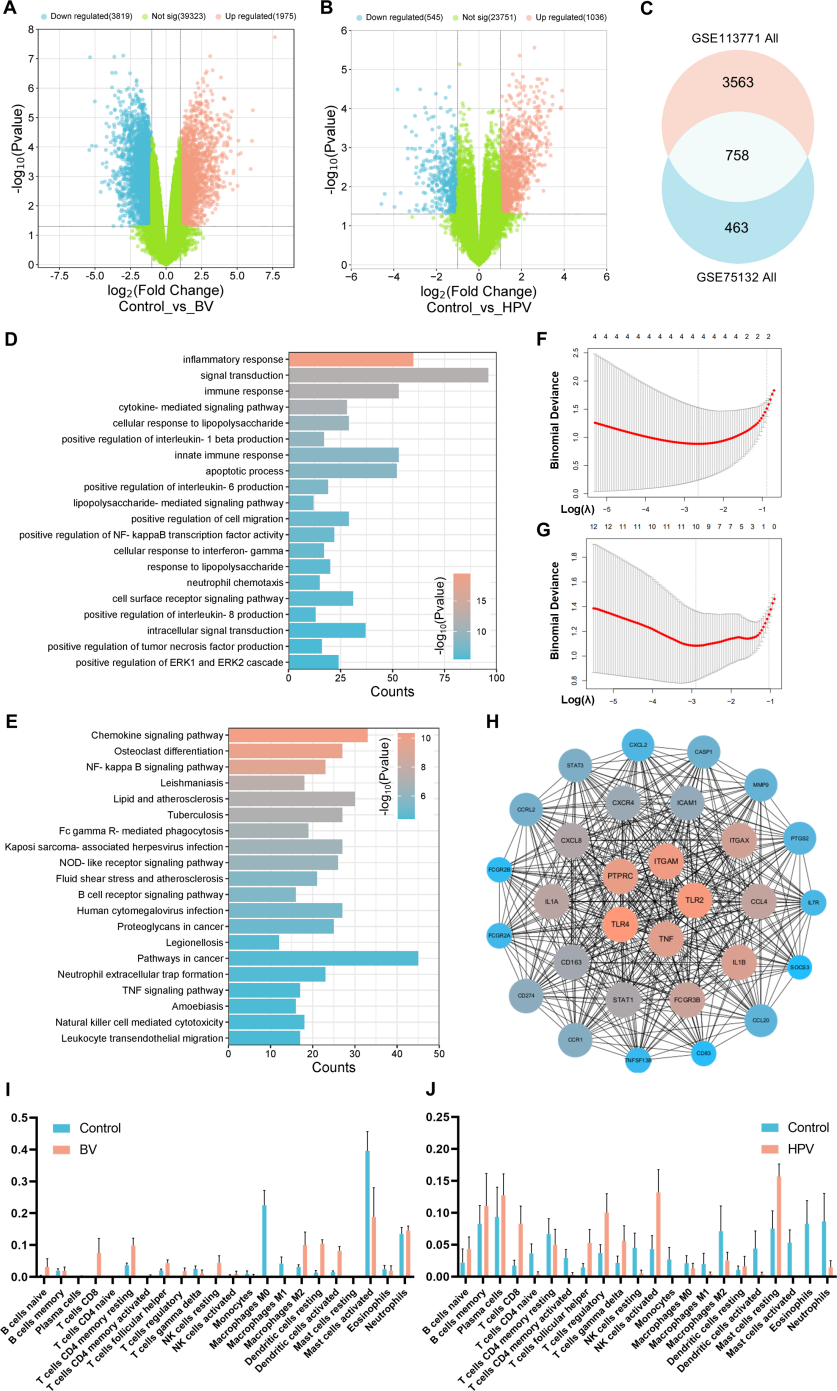
Shared characteristics of vaginal epithelial transcriptomics in BV and HPV infections. Volcano plot of **(A)** GSE113771 BV-related and **(B)** GSE75132 HPV-infected vaginal sample RNAseq; **(C)** Venn diagram of the co-DEGs between GSE113771 and GSE75132; Enrichment analysis of **(D)** GO biological processes and **(E)** KEGG signaling pathways based on co-DEGs; Lasso regression analysis of the set of disease targets for **(F)** BV or **(G)** HPV vaginal infections; **(H)** PPI analysis of the top 30 MCC scores in co-DEGs; Immune infiltrate analysis of **(I)** the BV-related vaginal sample (GSE113771) and **(J)** the HPV-infected vaginal sample (GSE75132) by CIBERSORT.

The BV-related vaginal sample RNA sequencing (RNAseq) database GSE113771 and the HPV-infected vaginal sample RNAseq database GSE75132 were screened from the GEO database (35, 36). GSE113771 analysis showed 1,975 genes up-regulated and 3,819 genes down-regulated in BV-related vaginal epithelium (**Figure 2A**). GSE75132 analysis showed 1,036 genes up-regulated and 545 genes down-regulated in epithelial cells after HPV infection (**Figure 2B**). Venn diagram represented a total of 758 co-differently expressed genes (co-DEGs) in both databases (**Figure 2C**). GO analysis showed that co-DEGs were mainly enriched in biological processes such as immune response, inflammatory cytokine regulation and receptor recognition (**Figure 2D**; **Supplementary Figure 4A, B**), and KEGG analysis showed that co-DEGs were mainly enriched in signaling pathways such as chemokines, NF-κB and FcγR mediated phagocytosis (**Figure 2E**).

Lasso regression analysis showed NFKBIA, PLAUR, SYK, and TLR2 as characteristic genes during BV, and 10 genes including CASP1, CD274, and CCL18, as characteristic genes during HPV infection (**Figure 2F, G**; **Supplementary Table 4**). These genes may be the core targets for regulating the disease process. Validation using Protein-Protein Interaction Networks (PPI) revealed the top 30 proteins with the highest maximum clique centrality (MCC) score, with TLR2, CASP1, and CD274 still labeled as core proteins (37–39) (**Figure 2H**). Proteins such as TNF-α, TLR4, IL-1β and CXCR4 have also been confirmed as immune-related genes (40, 41).

The immune cell composition was further analyzed by CIBERSORT (42). It revealed a significant infiltration of CD8^+^ T cells (*P* < 0.0001), resting DCs (*P* = 0.0033) and activated DCs (*P* = 0.0096) in BV-related vaginal epithelia (**Figure 2I**). Meanwhile, a significant infiltration of CD8^+^ T cells (*P* = 0.0204), Treg cells (*P* = 0.0470), activated NK cells (*P* = 0.0361) and activated mast cells (*P* = 0.0405) were observed in HPV-infected vaginal epithelia (**Figure 2J**). These results suggest that both innate and adaptive immune responses play crucial roles in BV/HPV co-infection, and they may jointly regulate disease progression through co-DEGs enriched signaling pathways and physiological processes.

### 3.3 Drug-disease target analysis

**Figure 3:**
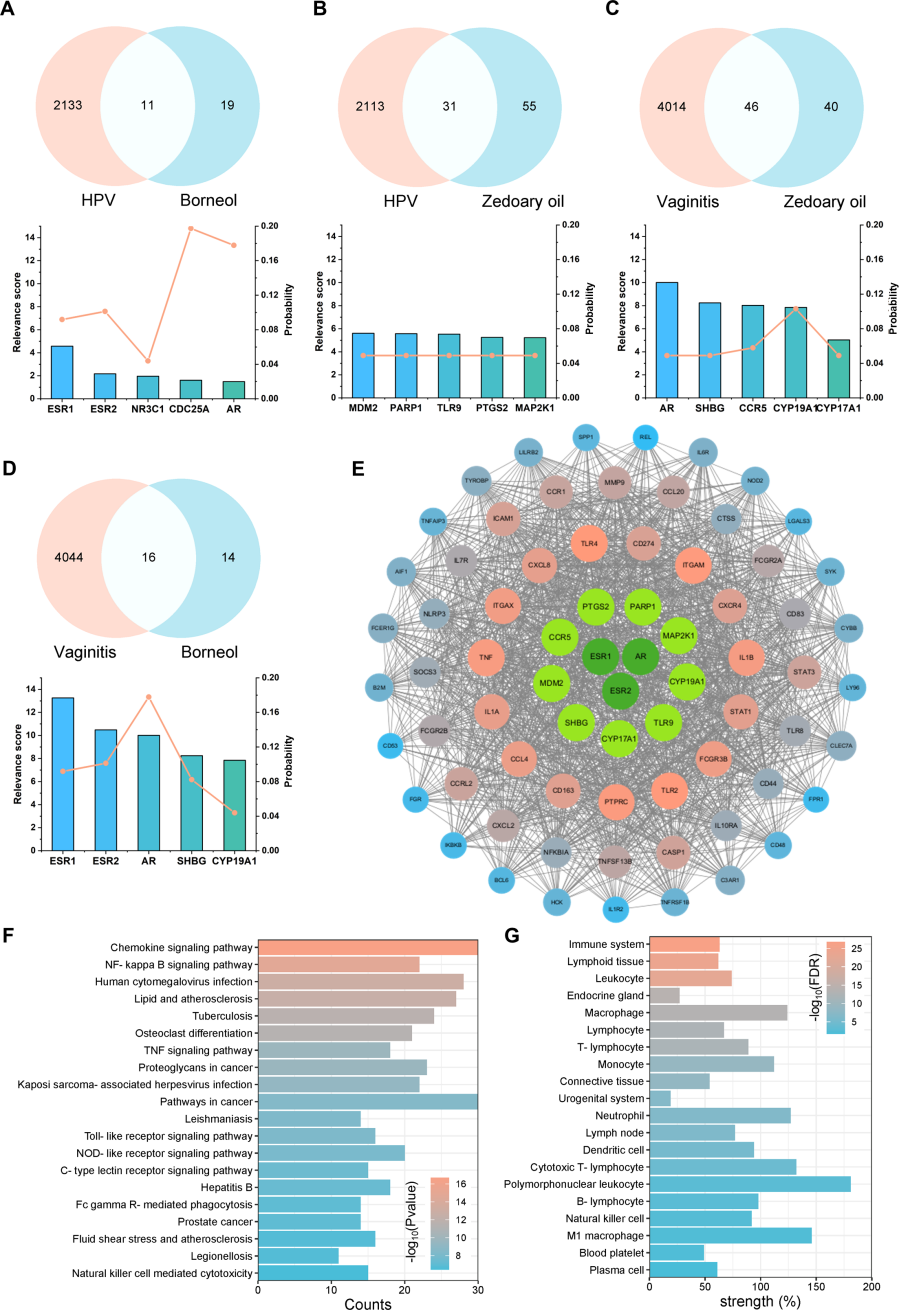
Drug-disease target and effect analysis of natural borneol with zedoary oil. Top five targets for disease relevance and target probability, along with Venn diagrams for: **(A)** natural borneol targets versus HPV disease targets, **(B)** zedoary oil targets versus HPV disease targets, **(C)** zedoary oil targets versus BV disease targets, **(D)** natural borneol targets versus BV disease targets; **(E)** PPI analysis, **(F)** GO biological process enrichment analysis, and **(G)** TISSUES enrichment analysis on the co-targets and the top 60 sub-targets with high MCC scores.

Utilizing TCMSP, TCMID, HIT databases, and related literature (43–45), we identified 35 potential active substances within natural borneol and zedoary oil (primary ingredients of Baofukang suppository) (**Supplementary Table 2**). The primary active ingredients were selected based on relative abundance, and their targets were predicted using SwissTargetPrediction (46, 47) (**Supplementary Table 3**). Subsequently, Targets of BV and HPV vaginal infections were collected by Genecards and DisGeNET (48, 49), and analyzed for their co-targets of action with the active compounds. The top five proteins in the disease relevance score among the co-targets and their probability are shown in **Figure 3A–D**. ESR1, ESR2 and AR are mainly associated with sex hormone action, but have also been shown to play a key role in immune regulation (50). Proteins such as TLR9, CCR5 and PTGS2 are also important components of innate immune activation (51, 52). Proteins directly interacting with co-targets (sub-targets) were screened from co-DEGs by PPI and the top 60 proteins with the highest MCC scores were demonstrated (**Figure 3E**). The results showed that proteins such as CD274, TLR4, and IL-1β still appeared as strongly associated molecules, suggesting that the drug efficacy involves immunomodulation. GO and KEGG analysis demonstrated the co-targets and sub-targets were enriched in signaling pathways such as NF-κB, IL-6 regulation and ERK1/2 (**Figure 3F**; **Supplementary Figure 6A-C**). TISSUES analysis showed the targets were enriched in physiological activities related to macrophages, T cells and neutrophils (53) (**Figure 3G**).

In summary, the co-targets and sub-targets of the primary active ingredients focused on regulating innate and adaptive immunity, encompassing pathogen recognition (e.g., TLR2, TLR9, CCR5, etc.), the secretion of inflammatory factors (e.g., IL-1β, IL-1α, TNF, etc.), T cell activation (e.g., ICAM-1, CD83, PTPRC, etc.), and other physiological processes.

Published literature has reported that in natural borneol, the relative abundance of borneol can reach 59%, while Dl-isoborneol can reach 38% (31) (**Supplementary Table 2**). Due to the high overlap in their disease-related target predictions, borneol was selected as the representative active component (**Supplementary Table 3**). In contrast, the active components of zedoary oil are more complex, primarily consisting of epicurzerenone, curzerene, curdione, curzerenone, and 1,8-cineole (30). However, the relative abundances of these components vary across different batches of zedoary oil, potentially due to variations in the growth environments of the extracted raw materials (30, 54, 55). Analysis of the disease-related target predictions indicated that curdione had the most abundant potential targets and its relative abundance can reach 20%, thereby making it the representative active component in zedoary oil (**Supplementary Table 2**, **3**). Although epicurzerenone and 1,8-cineole exhibited higher relative abundances, their predicted targets suggested significantly lower biological activities compared to those of curdione (**Supplementary Table 2**, **3**).

### 3.4 Innate immunomodulation capacity of curdione and borneol

**Figure 4:**
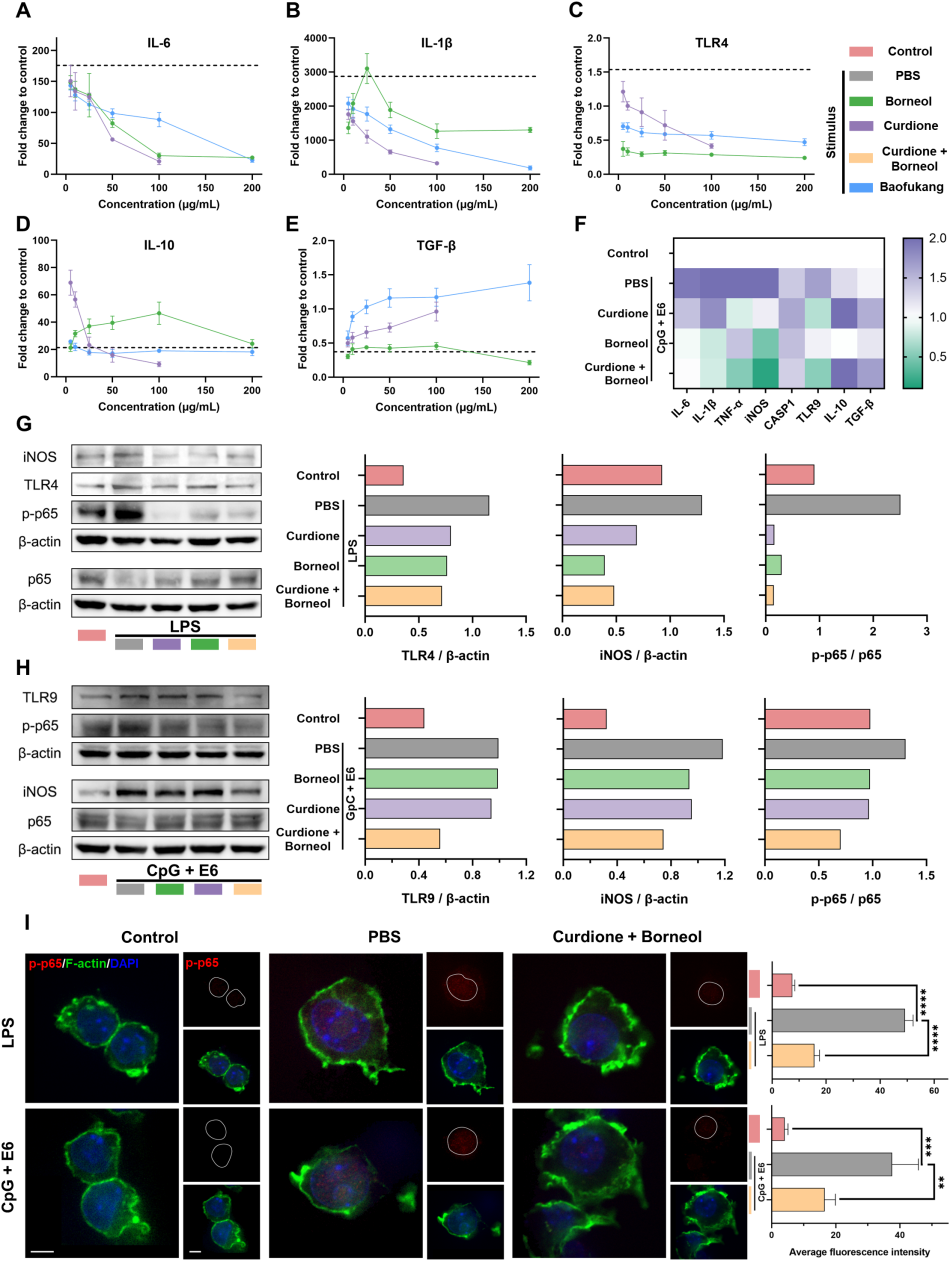
Curdione and borneol regulate innate immunity through the NF-κB signaling pathway. The mRNA expression levels of **(A)** IL-6, **(B)** IL-1β, **(C)** TLR4, **(D)** IL-10, and **(E)** TGF-β in the LPS-stimulated macrophage inflammatory activation model, following treatment with curdione, borneol, or Baofukang suppositories. (The dashed lines represent the mRNA expression levels of these cytokines in the model solely after LPS stimulation. n = 3); **(F)** Heatmap of mRNA expression levels of inflammation-associated proteins following treatment with curdione, borneol, or Baofukang suppositories in the CpG + E6-stimulated macrophage inflammatory activation model (n = 3); Expression levels of NF-κB p65, phosphorylated p65, and their upstream and downstream proteins after treatment with curdione and/or borneol in the **(G)** LPS-stimulated and **(H)** CpG+ E6-stimulated macrophage inflammatory activation models. (On the right, the ratios represent the quantification of gray values for protein expression.); **(I)** Images of phosphorylated p65 co-localized with nuclei (red: p-p65, green: F-actin, blue: DAPI, scale bar: 5 μm) in the model of inflammatory activation in macrophages stimulated by LPS or CpG + E6. Quantitative fluorescence statistics are also provided on the right (n = 3). Data are shown as mean ± SD; *P* values were calculated using a one-way ANOVA test, **P < 0.01, ***P < 0.001, ****P < 0.0001.

Based on the results of relative abundance and target prediction, we selected curdione and borneol as the representative active ingredients of Baofukang suppository to investigate their specific effects on immunity. In the classical inflammation model induced by LPS stimulation of Raw 264.7 cells, curdione, borneol and Baofukang suppositories dose-dependently inhibited the levels of IL-6, IL-1β and TLR4 mRNA expression (**Figure 4A-C**), while promoting the mRNA expression of IL-10 and TGF-β (**Figure 4D, E**). This suggests the main ingredient of Baofukang suppository may inhibit the excessive immune activation in BV/HPV co-infection. Based on the cytotoxicity and anti-inflammatory effects (**Supplementary Figure 2**), 100 μg/mL of curdione or borneol was selected for the subsequent experiments. Co-stimulation with CpG and HPV core protein E6 was used to simulate the inflammatory stimulus of HPV infection. Both curdione and borneol effectively inhibited the mRNA expression of pro-inflammatory cytokines while promoting the mRNA expression of anti-inflammatory cytokines in macrophages under this model (**Figure 4F**). There may exist a synergistic effect between curdione and borneol, further enhancing their anti-inflammatory effect.

NF-κB signaling pathway has been shown in shared disease characteristics (**Figure 2**) and drug-disease targets (**Figure 3**), and it is a key regulatory pathway of the inflammatory response (56). We have validated NF-κB signaling pathway in LPS-simulated and CpG + E6-simulated models, respectively. Curdione and borneol effectively inhibited the phosphorylation of p65 protein in both models, which in turn blocked the activation of NF-κB signaling pathway (**Figure 4G, H**). Meanwhile, the protein levels of upstream TLR4 (bacterial infection response) (57), TLR9 (viral infection response) (51) and downstream iNOS were also inhibited synchronously (**Figure 4G, H**). Upon activation, the p65 protein undergoes phosphorylation and nuclear translocation to bind to target molecules, thereby influencing the expression of inflammatory factors (58). The intracellular distribution of phosphorylated p65 (p-p65) was monitored by confocal microscopy, and the results showed curdione and borneol prevented the accumulation of p-p65 in the nucleus as expected (**Figure 4I**).

Overall, curdione and borneol, the primary active ingredients of Baofukang suppositories, can effectively modulate the expression of inflammatory factors such as IL-6 and IL-1β in macrophages by inhibiting the phosphorylation of NF-κB p65 protein and nuclear translocation, thus preventing the innate immune over-activation in BV/HPV co-infection.

### 3.5 Adaptive immunomodulation capacity of curdione and borneol

**Figure 5:**
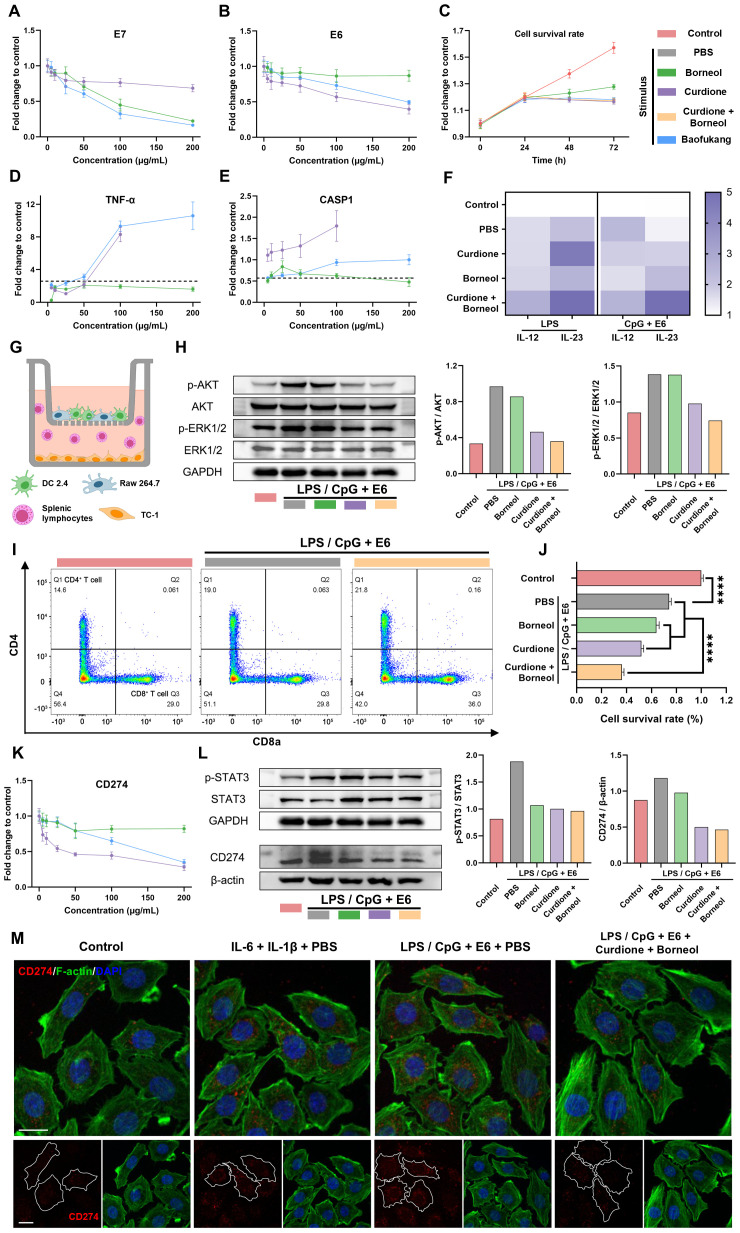
Curdione and borneol activate adaptive immunity and inhibit HPV immune escape through innate immunity. The expression levels of **(A)** E7 mRNA and **(B)** E6 mRNA in TC-1 cells at 1 d (n = 3) and **(C)** changes in TC-1 cell viability over 3 days after treatment with curdione, borneol or Baofukang suppositories (n = 4); The expression levels of **(D)** TNF-α mRNA and **(E)** CASP1 mRNA after treatment in the LPS-stimulated macrophage inflammatory activation model (n = 3); **(F)** IL-12 and IL-23 mRNA expression levels after treatment with curdione and/or borneol in the LPS-stimulated or CpG + E6-stimulated DCs inflammatory activation model (n = 3); **(G)** Transwell co-culture model of Raw 264.7 cells with DCs in the upper chamber and TC-1 with mouse splenic lymphocytes in the lower chamber; In the LPS/CpG + E6 co-stimulation co-culture model: **(H)** AKT, ERK1/2 and their phosphorylated protein expression levels in TC-1 cells (On the right, the ratios represent the quantification of gray values for protein expression), **(I)** The ratio of CD4^+^ to CD8^+^ T cell differentiation in splenic lymphocytes by flow cytometry (n = 4) and **(J)** the viability of TC-1 cell viability by MTT (n = 4); **(K)** CD274 mRNA expression levels in TC-1 cells following treatment with curdione, borneol, or Baofukang suppositories (n = 3); **(L)** STAT3, phosphorylated STAT3 and CD274 protein expression levels in TC-1 cells in the LPS/CpG + E6 co-stimulated co-culture model. (On the right, the ratios represent the quantification of gray values for protein expression.); **(M)** Confocal fluorescence imaging of CD274 in TC-1 cells in a co-culture model with different stimuli (red: CD274, green: F-actin, blue: DAPI, scale bar: 15 μm). Data are shown as mean ± SD; *P* values were calculated using a one-way ANOVA test, ****P < 0.0001.

Next, we investigated whether curdione and borneol could directly kill HPV-infected epithelial cells. TC-1 cells (mouse lung epithelial cells), which express HPV E6 and E7 proteins and exhibit similar biological behaviors to HPV-induced cervical cancer cells, were utilized to simulate HPV-infected vaginal epithelial cells *in vitro*. The qRT-PCR results showed that borneol and curdione significantly inhibited the mRNA expression of HPV E7 and E6, respectively (**Figure 5A, B**). The MTT cell viability assay confirmed that curdione and/or borneol, as well as Baofukang suppositories, could only inhibit the growth of TC-1 cells but did not directly kill them (**Figure 5C**).

While curdione and borneol inhibited inflammatory factors such as IL-6 and IL-1β expression in macrophages, TNF-α and CASP1 mRNA expression levels were up-regulated (**Figure 5D, E**), which are associated with anti-tumor and anti-viral responses (59, 60). Furthermore, in the LPS or CpG + E6 stimulated DC 2.4 cell model, both borneol and curdione up-regulated IL-12 and IL-23 mRNA expression (**Figure 5F**), which efficiently regulate T cell activation (61). A co-culture model was used to simulate the action of innate versus adaptive immunocyte crosstalk on TC-1 cells. In this model, mixed cultures of Raw 264.7 and DC 2.4 cells were placed in the upper chamber of the transwell system, and TC-1 cells were cultured in the lower chamber along with mouse splenic lymphocytes upon stimulation (**Figure 5G**). Western blot results indicated curdione effectively inhibited the phosphorylation levels of AKT and ERK1/2 proteins in TC-1 cells located in the lower chamber (**Figure 5H**). Activation of AKT and ERK1/2 has been reported to be associated with cell proliferation and precancerous lesions (62). Furthermore, flow cytometry analysis of splenic lymphocyte composition revealed that co-administration of curdione and borneol significantly increased the proportion of CD3^+^ CD8^+^ T cells (**Figure 5I**). MTT assays demonstrated curdione + borneol combined with splenic lymphocytes exhibited a significant killing effect on TC-1 cells in the co-culture model (**Figure 5J**). These results suggest that curdione and borneol may modulate the crosstalk between innate and adaptive immune cells, thereby achieving the killing of HPV-infected cells via T cell activation.

It has been reported that IL-6 and IL-1β can stimulate tumor cells to express CD274 (PD-L1) proteins via activating the STAT3 signaling pathway, thereby facilitating tumor cell immune escape (22, 63). Considering the observed phenotype of curdione and borneol inhibiting macrophage secretion of IL-6 and IL-1β, we further explored the CD274 expression levels of TC-1 cells in a co-culture model. The qRT-PCR results showed that the level of CD274 mRNA expression in TC-1 cells was inhibited by curdione and borneol (**Figure 5K**). Western blot results showed the inflammatory environment, induced by the combined stimulation of macrophages and DCs with LPS/CpG + E6, promoted the phosphorylation of STAT3 protein and subsequently induced the expression of CD274 in TC-1 cells. Notably, this effect was reversed by both curdione and borneol (**Figure 5L**). Confocal microscopy results revealed that both direct stimulation with IL-6 and IL-1β, as well as combined LPS/CpG + E6 stimulation in the co-culture model, induced CD274 protein expression in TC-1 cells. The high CD274 expression observed in the co-culture stimulation model was inhibited by curdione + borneol (**Figure 5M**). The above results suggest that curdione and borneol effectively blocked STAT3 phosphorylation-mediated CD274 upregulation in TC-1 cells by inhibiting macrophage secretion of IL-6 and IL-1β. This may enhance the recognition and killing of HPV-infected cells by CD3^+^ CD8^+^ T cells. These findings also explain the clinical phenomenon wherein HPV is difficult to eradicate in cases of BV/HPV co-infection.

### 3.6 Curdione and borneol eliminate HPV-infected epithelial cells *in vivo*

**Figure 6:**
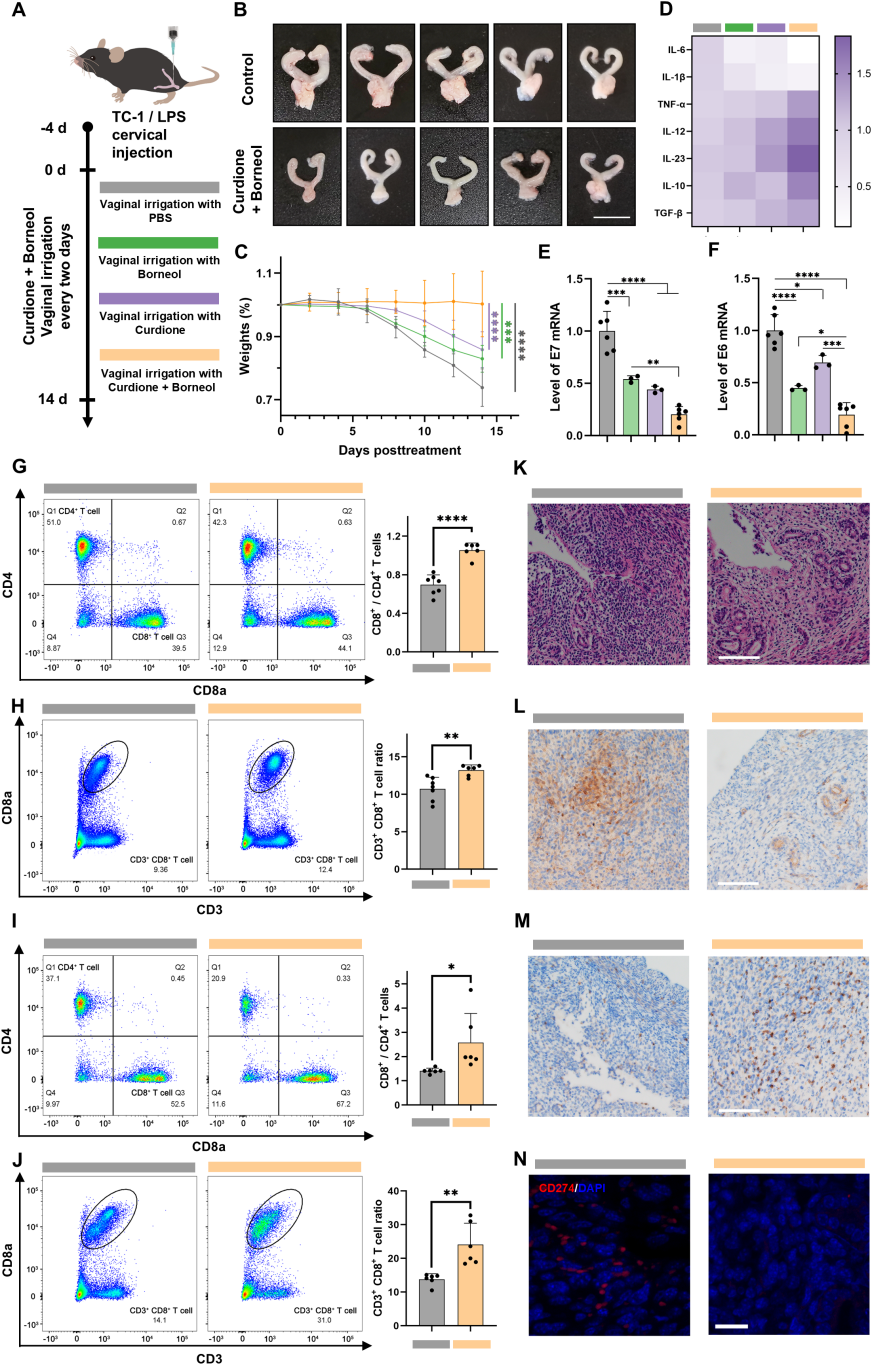
Curdione and borneol eliminate HPV-infected epithelial cells in the TC-1/LPS cervical *in situ* injection model. **(A)** Schematic diagram of TC-1/LPS cervical *in situ* injection model construction; In the TC-1/LPS cervical *in situ* injection model after 14 d of PBS (Control group), curdione, borneol or curdione + borneol vaginal lavage treatment (Combined treatment group): **(B)** Representative images of mouse uterus (n = 5), **(C)** weight change (n = 5), mRNA expression levels of **(D)** inflammation-related proteins (n = 6), **(E)** HPV E6 proteins and **(F)** HPV E7 proteins (n = 3), **(G)** the ratio of CD4^+^ to CD8^+^ T cell differentiation and **(H)** the ratio of CD3^+^ CD8^+^ T cell in the spleen (n = 6), **(I)** the ratio of CD4^+^ to CD8^+^ infiltrated T cell differentiation and **(J)** the ratio of CD3^+^ CD8^+^ infiltrating T cells in the cervix and vagina (n = 6); **(K)** H&E staining (scale bar: 100 μm, n = 3), **(L)** immunohistochemical staining for IL-6 (scale bar: 100 μm, n = 3), **(M)** immunohistochemical staining for CD8a (scale bar: 100 μm, n = 3), and **(N)** immunofluorescence staining for CD274 (red: CD274, blue: DAPI, scale bar: 20 μm, n = 3) of the cervix and vagina from the TC-1/LPS cervical *in situ* injection model. Data are shown as mean ± SD; *P* values were calculated using Student’s t-test, *P < 0.05, **P < 0.01, ***P < 0.001, ****P < 0.0001.

Finally, we investigated whether curdione and borneol could reproduce their immunomodulatory effects *in vivo* and eliminate HPV-infected epithelial cells in HPV/BV co-infections. Given that HPV exclusively infects humans, we employed TC-1 cells in combination with LPS orthotopic injection into the cervix of C57/B6 mice to mimic HPV/BV co-infections. Four days after the TC-1/LPS orthotopic injection, mice received intravaginal lavages with 100 μg/mL of curdione + borneol (combined treatment group) or PBS (control group) every two days for a total of seven treatments (**Figure 6A**). If curdione + borneol effectively promotes the clearance of HPV-infected epithelial cells in HPV/BV co-infections, it could prevent the formation of *in situ* HPV-associated cervical tumors derived from TC-1 cells. After 14 days, mice were euthanized, and their uteruses were harvested. In the control group, all TC-1 cells formed orthotopic tumors (11/11), whereas only 22.22% of mice in the curdione + borneol treatment group (2/9) developed orthotopic tumors (**Figure 6B**, **Supplementary Figure S12A, D**). When treated with curdione (3/5) or borneol (4/6) alone, the therapeutic effect was inferior to that of combination therapy (**Supplementary Figure S12B, C**). The cervical and vaginal tissues of the combined treatment group exhibited lower mRNA expression levels of IL-6, IL-1β and CD274, alongside higher mRNA expression levels of IL-10 and TGF-β (**Figure 6D**; **Supplementary Figure S13**). Mice in the treatment group maintained their weight, while mice in the control group experienced rapid weight loss due to tumor formation and vaginal inflammation (**Figure 6C**). Moreover, the mRNA expression levels of E6 and E7 in the cervical and vaginal tissues were significantly reduced after curdione + borneol treatment, reflecting the elimination of TC-1 cells in the treatment group (**Figure 6E, F**). While curdione or borneol alone exhibited some anti-inflammatory and antiviral effects, the therapeutic outcomes were less superior to combination therapy, and the mRNA levels of CD274 were also higher.

Further research was conducted to investigate the therapeutic effects of combination therapy. Flow cytometry analysis of spleens from the combined treatment group showed a significant increase in both the number of CD3^+^ CD8^+^ T cells and the ratio of CD3^+^ CD8^+^ to CD3^+^ CD4^+^ T cells (**Figure 6G, H**; **Supplementary Figure 14**, **15**). Furthermore, an elevation in the mRNA expression of TNF-α, IL-12, and IL-23 was detected in cervical and vaginal tissues (**Figure 6D**; **Supplementary Figure 13**). These results are consistent with the conclusion that curdione + borneol modulates innate-adaptive immune crosstalk *in vitro*, enhancing cytotoxic T cell activation. Analogously, flow cytometry of cervical tissues mirrored splenic findings, revealing an increase in both the quantity and proportion of infiltrating cytotoxic T cells (**Figures 6I, J** **;** **Supplementary Figure 16**, **17**).

H&E staining revealed a more normalized tissue and glandular architecture in the cervixes of the curdione + borneol treatment group, in contrast to the disorganized structure observed in the control group (**Figure 6K**; **Supplementary Figure 18**). Immunohistochemical staining demonstrated lower IL-6 expression and a higher infiltration of CD8a^+^ cells in the combined treatment group (**Figure 6L, M**; **Supplementary Figure 19**, **20**). Notably, CD274 protein expression was significantly lower in the combined treatment group compared to the control (**Figure 6N**; **Supplementary Figure 21**), which explains the pronounced infiltration of CD3^+^ CD8^+^ T cells in the cervical tissues of the combined treatment group and correlates with the reduced inflammation in this group. The primary active components of Baofukang suppository, curdione + borneol, demonstrated their ability to modulate both innate and adaptive immunity in the mouse model of HPV/BV co-infections. While activating cytotoxic T cells, they suppressed the secretion of IL-6 and IL-1β, thereby blocking the STAT3-CD274 axis-mediated immune evasion and enhancing T cell recognition and infiltration. This effective clearance of HPV-infected epithelial cells under mixed infection conditions facilitated the cure of persistent HPV infections.

## 4 Discussion

HPV has garnered significant academic attention as a widespread and cancer-causing disease of the reproductive system (1). Following the introduction of HPV vaccines, the prevention of HPV has become a reality (64). However, for patients with persistent high-risk HPV infections, there is currently no specific drug that can provide direct relief (65). The etiology of persistent HPV infections is multifaceted, encompassing disruptions in the vaginal microenvironment, compromised immunity, and repeated HPV exposure (9, 10, 64). Compared to healthy individuals, these patients have a weakened vaginal defense, making them more susceptible to other pathogens, especially bacterial infections (2). Furthermore, HPV susceptibility in BV patients has been reported (5). The intricate interplay between HPV and BV, regardless of whether HPV precedes BV or vice versa, underscores the pivotal role of the intravaginal microenvironment and immune response in BV/HPV co-infections.

Imiquimod holds significant promise as a therapeutic option for the management of persistent HPV infections (65). As an immunomodulatory agent, it can effectively activate macrophages, thereby enhancing T-cell-mediated cytotoxicity. For BV patients, antibiotics and anti-inflammatories have even greater significance (5). An excessive inflammatory response can damage vaginal tissues and hinder the recolonization of beneficial probiotics such as lactobacilli (64). In cases of BV/HPV co-infections, clinicians require more types of medications to administer, aiming to modulate the intricate and robust immune responses involved.

Baofukang suppository, formulated from traditional Chinese medicine extracts, has been extensively utilized in the treatment of BV in China and has demonstrated its efficacy in long-term clinical practice (28). Recently, several cohort studies have discovered its active role in the management of HPV infection (27–29). Therefore, we designed and tracked a retrospective cohort of patients with bacterial mixed HPV infection, discovering that Baofukang suppository not only improved the vaginal microenvironment but also facilitated HPV clearance. However, Baofukang suppository did not exhibit bactericidal activity against Gardnerella vaginalis at non-cytotoxic concentrations, suggesting that its therapeutic effects are mediated by alternative mechanisms.

There is now growing evidence that both innate and adaptive immunity play significant roles in HPV infection and cervical carcinogenesis (11–13). To better understand the disease characteristics of BV/HPV co-infection, we utilized the GEO database and bioinformatics analysis. Innate immune-related molecules such as TLR4, TLR2, CXCR4 and adaptive immune-related molecules such as CD274, ICAM1, PTGS2 were identified as nodal molecules. Also, target analysis based on network pharmacology revealed that these immune-related molecules were widely present among the sub-targets. Notably, the multiple enrichments of the NF-κB signaling pathway suggest that Baofukang Suppository may treat BV/HPV co-infection by modulating immune responses.

The NF-κB signaling pathway is one of the key mechanisms of innate immune activation and can effectively regulate the secretion of inflammatory factors (56). Based on the drug targets and component ratios, we selected curdione and borneol as the core active ingredients of Baofukang suppository for further research. Similar to plant-derived sesquiterpenoids like parthenolide, curdione and borneol effectively inhibited the phosphorylation and nuclear translocation of NF-κB p65. They suppressed the expression levels of inflammation-related mRNAs, including IL-6, IL-1β, and TLR4, in macrophage models stimulated with LPS or CpG + E6. In the LPS/TC-1 cervical *in situ* injection model, the expression of inflammatory factors in vaginal and cervical tissues was also effectively suppressed. Curdione and borneol effectively inhibit the overactivated inflammatory response under BV/HPV co-infection, thereby protecting vaginal and cervical tissues.

Interestingly, we found that curdione and borneol induced macrophages to express TNF-α and CASP1, and also induced DCs to upregulate the mRNA expression levels of IL-12 and IL-23. IL-12 and IL-23 act on both innate and adaptive immunity, including natural killer (NK) cells and CD8^+^ T cells (61). We further validated the crosstalk between innate and adaptive immune cells in the co-culture model. Curdione and borneol effectively promoted an increase in the proportion of CD8^+^ T cells by stimulating macrophages and DCs. In the mouse model, the proportion of CD3^+^ CD8^+^ splenic lymphocytes was also significantly increased in the treated group. Thus, curdione and borneol promote the effective activation of adaptive immunity by modulating the crosstalk among immune cells.

IL-6 and IL-1β can enhance CD274 expression in tumor cells by activating the JAK2/STAT3 signaling pathway, making it difficult to be recognized by T cells and facilitating tumor immune evasion (22, 63). Previous research has reported high expression of CD274 in tumor tissues of HPV-related cervical cancer patients, and the HPV proteins may be involved in CD274 regulation (64). We have noticed that STAT3 and CD274 have repeatedly been identified as characteristic and sub-target proteins in PPI and Lasso analyses. As previously mentioned, curdione and borneol have been proven to inhibit the phosphorylation and nuclear translocation of NF-κB p65. As a classic pathway regulating the transcriptional expression of inflammatory cytokines, inhibition of the NF-κB signaling pathway significantly reduces the secretion of IL-6 and IL-1β by macrophages (65, 66). We hypothesized that curdione and borneol can enhance the recognition and killing functions of CLTs by reducing IL-6 and IL-1β levels in the microenvironment, thereby preventing the upregulation of CD274 in infected epithelial cells. Exploring this mechanism in the co-culture model, curdione and borneol effectively inhibited STAT3 protein phosphorylation and CD274 expression. Meanwhile, in the cervix of the treated group, the CD274 expression was significantly decreased and the infiltration of CD3^+^ CD8^+^ T cells was more pronounced. The IL-6/IL-1β-STAT3-CD274 axis-mediated immune escape during cervical cancer pathology may also exist during HPV infection, and curdione and borneol can block this process through modulating inflammatory factors.

In the co-culture model, the phosphorylation levels of AKT and ERK in TC-1 cells were inhibited by curdione. Previous literature has reported that IL-6 activates the PI3K/AKT and MAPK/ERK signaling pathways by binding to IL-6R on target cells (67). As a ubiquitous and conserved signaling pathway, activated PI3K/AKT effectively enhances cellular metabolic synthesis and proliferation (68). Meanwhile, ERK, a core regulator of various cellular activities, broadly influences cell migration, proliferation, and differentiation upon its phosphorylation (69). Hyperactivation of AKT and ERK leads to uncontrolled cell proliferation and cancer initiation. Curdione has been reported to inhibit the activation of ERK in mouse lung pyemia and to suppress AKT phosphorylation in breast cancer (70, 71). In the co-culture model, curdione not only blocked the activation of AKT and ERK in TC-1 cells by inhibiting IL-6 from macrophages but also directly interacted with TC-1 cells to exert inhibitory effects. The synergistic effect of borneol was related to the inhibiting of inflammatory cytokines. The downregulation of AKT and ERK phosphorylation suppressed the proliferation of TC-1 cells, providing an alternative explanation for the combined therapeutic efficacy of curdione and borneol. Additionally, this finding elucidated the molecular mechanism underlying the progression of HPV facilitated by the high inflammatory levels associated with BV co-infection.

Our study also has some limitations. Firstly, the existing drug target prediction systems and disease target databases remain incomplete, and the relevant data in the GEO database is relatively scarce, potentially leading to an incomplete identification of targets. Secondly, since HPV exclusively infects humans, there is no ideal mouse model to represent HPV infection. The use of TC-1 cervical *in situ* injections to mimic HPV infection does not fully capture the disease state in the pre-intermediate stages of HPV infection, thereby limiting the applicability of our findings to the advanced stages of HPV infection and cervical precancerous lesions. Nevertheless, our work first demonstrated the potential mechanisms of Baofukang suppository for the treatment of BV/HPV co-infection, and more studies are required to be explored in the future.

## 5 Conclusions

In summary, this study validated the therapeutic efficacy of Baofukang suppository against BV/HPV co-infection and demonstrated that curdione and borneol can activate adaptive immunity by modulating innate immunity. We also propose that there may be an IL-6/IL-1β-STAT3-CD274 axis-mediated immune escape occurring during HPV infection, and that this process could potentially be blocked by inhibiting excessive inflammation. Overall, this work firstly provides a theoretical basis for the treatment of BV/HPV co-infection with Baofukang suppository, which holds promise as an effective therapeutic option for bacterial mixed HPV infections.

## Data Availability

The raw data supporting the conclusions of this article will be made available by the authors, without undue reservation.
