# Supplementary material for: Curdione combined with borneol treats bacterial mixed HPV infection by regulating the crosstalk among immune cells
Source: Front Immunol. 2025 Jan 22;16:1503355. doi: 10.3389/fimmu.2025.1503355 (PMC11794296; doi:10.3389/fimmu.2025.1503355)
Supplement: Supplementary file 2 [file Table2.docx]

Supplementary Material

# Supplementary Tables

# Supplementary.Table.1. Basic information of the cohort of 205 patients with HPV vaginal infection in a single center.

|  |  | All | Control | Treat | P.value |
| --- | --- | --- | --- | --- | --- |
| Sample |  | 205 | 104 | 101 |  |
| Age |  | 41.16 | 41.87 | 40.44 | 0.262 |
| Pregnancy frequency |  | 1.78 | 1.78 | 1.77 | 0.951 |
| Delivery frequency |  | 1.31 | 1.35 | 1.27 | 0.328 |
| First gestational age |  | 23.45 | 23.3 | 23.61 | 0.186 |
| Recent sexual frequency (times/week) |  | 1.76 | 1.72 | 1.8 | 0.599 |
| Microbial diversity | Wellness | 33 | 16 | 17 | 0.778 |
|  | Abnormal | 172 | 88 | 84 |  |
| Microbial density | Wellness | 188 | 95 | 93 | 0.849 |
|  | Abnormal | 17 | 9 | 8 |  |
| Nugent level | Ⅰ | 12 | 8 | 4 | 0.177 |
|  | Ⅱ | 178 | 90 | 88 |  |
|  | Ⅲ | 15 | 6 | 9 |  |
| AV level | Ⅰ | 175 | 90 | 85 | 0.631 |
|  | Ⅱ | 30 | 14 | 16 |  |
| Trichomonas | - | 203 | 103 | 100 | 0.983 |
|  | + | 2 | 1 | 1 |  |
| Candida | - | 170 | 86 | 84 | 0.928 |
|  | + | 35 | 18 | 17 |  |

# Supplementary.Table.2. Analysis of the main active components of natural borneol and zedoary oil.

| Component | Relative Abundance | Canonical SMILES | Lipinski | Ghose | Veber | Egan | Muegge | Bioavailability Score |
| --- | --- | --- | --- | --- | --- | --- | --- | --- |
| **Natural borneol：** | | |  |  |  |  |  |  |
| Borneol | 59.78–58.93% | CC1(C2CCC1(C(C2)O)C)C | Yes | MW<160 | Yes | Yes | MW<200, Heteroatoms<2 | 0.55 |
| Dl-Isoborneol | 38.98–37.52% | CC1(C2CCC1(C(C2)O)C)C | Yes | MW<160 | Yes | Yes | MW<200, Heteroatoms<2 | 0.55 |
| Camphor | 2.70–2.09%. | CC1(C2CCC1(C(=O)C2)C)C | Yes | MW<160 | Yes | Yes | MW<200, Heteroatoms<2 | 0.55 |
| Caryophellene | N.S. | CC1=CCCC(=C)C2CC(C2CC1)(C)C | Yes; MLOGP>4.15 | Yes | Yes | Yes | Heteroatoms<2 | 0.55 |
| Oleanolic Acid | N.S. | CC1(CCC2(CCC3(C(=CCC4C3(CCC5C4(CCC(C5(C)C)O)C)C)C2C1)C)C(=O)O)C | Yes; MLOGP>4.15 | WLOGP>5.6, MR>130, #atoms>70 | Yes | WLOGP>5.88 | XLOGP3>5 | 0.85 |
| Humulene | N.S. | CC1=CCC(C=CCC(=CCC1)C)(C)C | Yes; MLOGP>4.15 | Yes | Yes | Yes | Heteroatoms<2 | 0.55 |
| Alphitolic Acid | N.S. | CC(=C)C1CCC2(C1C3CCC4C(C3(CC2)C)(CCC5C4(CC(C(C5(C)C)O)O)C)C)C(=O)O | Yes; violation: MLOGP>4.15 | WLOGP>5.6, MR>130, #atoms>70 | Yes | WLOGP>5.88 | XLOGP3>5 | 0.56 |
| Asiatic Acid | N.S. | CC1CCC2(CCC3(C(=CCC4C3(CCC5C4(CC(C(C5(C)CO)O)O)C)C)C2C1C)C)C(=O)O | Yes | MW>480, MR>130, #atoms>70 | Yes | Yes | XLOGP3>5 | 0.56 |
| Dipterocarpol | N.S. | CC(=CCCC(C)(C1CCC2(C1CCC3C2(CCC4C3(CCC(=O)C4(C)C)C)C)C)O)C | Yes; MLOGP>4.15 | WLOGP>5.6, MR>130, #atoms>70 | Yes | WLOGP>5.88 | XLOGP3>5 | 0.55 |
| Dryobalanone | N.S. | CC(=CCCC(CO)(C1CCC2(C1CCC3C2(CCC4C3(CCC(=O)C4(C)C)C)C)C)O)C | Yes; MLOGP>4.15 | WLOGP>5.6, MR>130, #atoms>70 | Yes | WLOGP>5.88 | XLOGP3>5 | 0.55 |
| Elemene | N.S. | CC(=C)C1CCC(C(C1)C(=C)C)(C)C=C | Yes; MLOGP>4.15 | Yes | Yes | Yes | XLOGP3>5, Heteroatoms<2 | 0.55 |
| Erythrodiol | N.S. | CC1(CCC2(CCC3(C(=CCC4C3(CCC5C4(CCC(C5(C)C)O)C)C)C2C1)C)CO)C | Yes; MLOGP>4.15 | WLOGP>5.6, MR>130, #atoms>70 | Yes | WLOGP>5.88 | XLOGP3>5 | 0.55 |
| Bronyl acetate | N.S. | CC(=O)OC1CC2CCC1(C2(C)C)C | Yes; | Yes | Yes | Yes | MW<200 | 0.55 |
| **Zedoary oil:** | | |  |  |  |  |  |  |
| Epicurzerenone | 19.0–46.6% | CC1=COC2=C1C(=O)C(C(C2)(C)C=C)C(=C)C | Yes | Yes | Yes | Yes | Yes | 0.55 |
| Curzerene | ~10.4% | CC1=COC2=C1CC(C(C2)(C)C=C)C(=C)C | Yes | Yes | Yes | Yes | Heteroatoms<2 | 0.55 |
| Curdione | 7.0–19.6% | CC1CCC=C(CC(=O)C(CC1=O)C(C)C)C | Yes | Yes | Yes | Yes | Yes | 0.55 |
| Curzerenone | 7.0–19.6% | CC1=COC2=C1C(=O)C(C(C2)(C)C=C)C(=C)C | Yes | Yes | Yes | Yes | Yes | 0.55 |
| 1,8-cineole | 18.5–40.8% | CC1(C2CCC(O1)(CC2)C)C | Yes | MW<160 | Yes | Yes | MW<200, Heteroatoms<2 | 0.55 |
| β-sesquiphellandrene | ~21.5% | CC(CCC=C(C)C)C1CCC(=C)C=C1 | Yes; MLOGP>4.15 | Yes | Yes | Yes | XLOGP3>5, Heteroatoms<2 | 0.55 |
| p-cymene | N.S. | CC1=CC=C(C=C1)C(C)C | Yes; MLOGP>4.15 | MW<160 | Yes | Yes | MW<200, Heteroatoms<2 | 0.55 |
| Curcumenene | N.S. | CC1=CC=C(C=C1)C(C)CCC=C(C)C | Yes; MLOGP>4.15 | Yes | Yes | Yes | XLOGP3>5, Heteroatoms<2 | 0.55 |
| α-phellandrene | N.S. | CC1=CCC(C=C1)C(C)C | Yes | MW<160 | Yes | Yes | MW<200, Heteroatoms<2 | 0.55 |
| curcumol | N.S. | CC1CCC2C13CC(C(O3)(CC2=C)O)C(C)C | Yes | Yes | Yes | Yes | Yes | 0.55 |
| β-elemene | N.S. | CC(C)C1=CC(C(CC1)(C)C=C)C(=C)C | Yes; MLOGP>4.15 | Yes | Yes | Yes | XLOGP3>5, Heteroatoms<2 | 0.55 |
| α-pinene | N.S. | CC1=CCC2CC1C2(C)C | Yes; MLOGP>4.15 | MW<160 | Yes | Yes | MW<200, Heteroatoms<2 | 0.55 |
| Tetramethylpyrazine | N.S. | CC1=C(N=C(C(=N1)C)C)C | Yes | MW<160 | Yes | Yes | MW<200 | 0.55 |

# Supplementary.Table.3. Drug targets of the main active components of natural borneol and zedoary oil.

| Component | Targets |
| --- | --- |
| **Natural borneol：** | |
| Borneol | CA2, CA1, CA4, TRPM8, CDC25A, CDC25B, NR1I3, AR, NR1H4, GPBAR1, SHH, ESR2, ESR1, UGT2B7, SHBG, POLA1, NPC1L1, HSD11B1, NR1H3, G6PD, GABBR1, NR3C1, DRD2, SIGMAR1, GABRB2, GABRG2, GABRA2, AKR1B10, POLB, CYP19A1 |
| Dl-Isoborneol | CA2, CA1, CA4, TRPM8, CDC25A, CDC25B, NR1I3, AR, NR1H4, GPBAR1, SHH, ESR2, ESR1, UGT2B7, SHBG, POLA1, NPC1L1, HSD11B1, NR1H3, G6PD, GABBR1, NR3C1, DRD2, SIGMAR1, GABRB2, GABRG2, GABRA2, AKR1B10, POLB, CYP19A1 |
| Camphor | NR1I3, CYP19A1, CA2, CA1, CA4, AR, CES1, CES2, ADH1A, ADH1C, SERPINA6, SHBG, G6PD, GABBR1, CTSK, VDR, NPC1L1, NR1H4, GABRB2, GABRG2, GABRA2, CDC25A, GPBAR1, AKR1B10, POLB |
| **Zedoary oil:** | |
| Epicurzerenone | SLC6A4, SLC6A3, PTGS2, PABPC1, HSD17B2, NLRP3, EPHX1, PREP, CTSK, CTSL, CTSB, MDM2, HCRTR1, NOS2, PTGS1, AR, CDC25B, HTR6 |
| Curzerene | DRD4 |
| Curdione | CYP19A1, CTSD,PTGS1, CCR5, TRPV1, FAAH, MTNR1A, MTNR1B, CTSK, CTSL, CTSB, AR, SIGMAR1, CTSS, P2RX7, ACE, CTSC, PSEN2, PSENEN, NCSTN, APH1A, PSEN1, APH1B, CYP17A1, GABRB3, GABRA3, GABRG2, GABRA1, GABRA5, GABRA2, GABRA6, PARP1, EPHX2, SERPINA6, SHBG, NR1I2, FABP1, CYP51A1, PIN1, FAP, CHRM1, MAOB, MC4R, CHRM4, CHRM5, F2, PRSS1, CHRM3, CDC25A, SRD5A1, NQO2, PDE7A, GPR139, PPARG, GABRA1, GABRB2, GABRG2, BRD4, MAP2K1, HTR2B, EPHX1, PDE4B, PPARA, FABP5, PPARD, SRD5A2, HMOX1, MPO, HSD17B3, KCNA5, FKBP1A, HTR6, GRM5, TLR9, CYP11B2, TNKS2, SIRT2 |
| Curzerenone | SLC6A4, SLC6A3, PTGS2, PABPC1, HSD17B2, NLRP3, EPHX1, PREP, CTSK, CTSL, CTSB, MDM2, HCRTR1, NOS2, PTGS1, AR, CDC25B, HTR6 |
| 1,8-cineole | CYP19A1, SHH, CYP51A1, ACHE |

# Supplementary.Table.4. Characteristic Gene by Lasso regression analysis.

| Lasso regression analysis | Characteristic Gene |
| --- | --- |
| BV (GSE113771) | NFKBIA, PLAUR, SYK, TLR2 |
| HPV (GSE75132) | CASP1, CCL18, CD274, DEFB1, DLG5, GBP5, KRT18, PDE4B, SDC1, SPP1 |

# Supplementary.Table.5. The sequences of qRT-PCR primers.

| Genes | Forward primer | Reverse primer |
| --- | --- | --- |
| HPV16-E6 (1) | AATGTGTGTACTGCAAGCAAC | GACACAGTGGCTTTTGACAGT |
| HPV16-E7 (1) | ATGGAGATACACCTACATTGC | CACAACCGAAGCGTAGAGTCA |
| Mouse-CD274 | AGCCTGGAACAACGGACATT | CTGCTAAGCCAGGAACCCTC |
| Mouse-CASP1 | ACTGACTGGGACCCTCAAGT | GCAAGACGTGTACGAGTGGT |
| Mouse-TLR9 | CCATGGACCTGTCTCGGAAC | ACTGAGAGCCATTGACAGCC |
| Mouse-TLR4 | CGCTGCCACCAGTTACAGAT | TCTGATCCATGCATTGGTAGGT |
| Mouse-IL-6 | ACAAGTCCGGAGAGGAGACT | GAATTGCCATTGCACAACTCT |
| Mouse-IL-1β | AGCTTCAGGCAGGCAGTATC | CGTCACACACCAGCAGGTTA |
| Mouse-TNF-α | GCACAGAAAGCATGATCCGC | AACTGATGAGAGGGAGGCCA |
| Mouse-iNOS | CCCTTCAATGGTTGGTACATGG | ACATTGATCTCCGTGACAGCC |
| Mouse-IL-10 | CAGTACAGCCGGGAAGACAAT | TTGGCAACCCAAGTAACCCT |
| Mouse-TGF-β | AGGGCTACCATGCCAACTTC | CCACGTAGTAGACGATGGGC |
| Mouse-IL-12a | TGGATCTGAGCTGGACCCTT | GTTGGAACGCTGACCATAGAGA |
| Mouse-IL-23 | CCAGCGGGACATATGAATCTAC | TGTCCTTGAGTCCTTGTGGG |

# Supplementary Figures


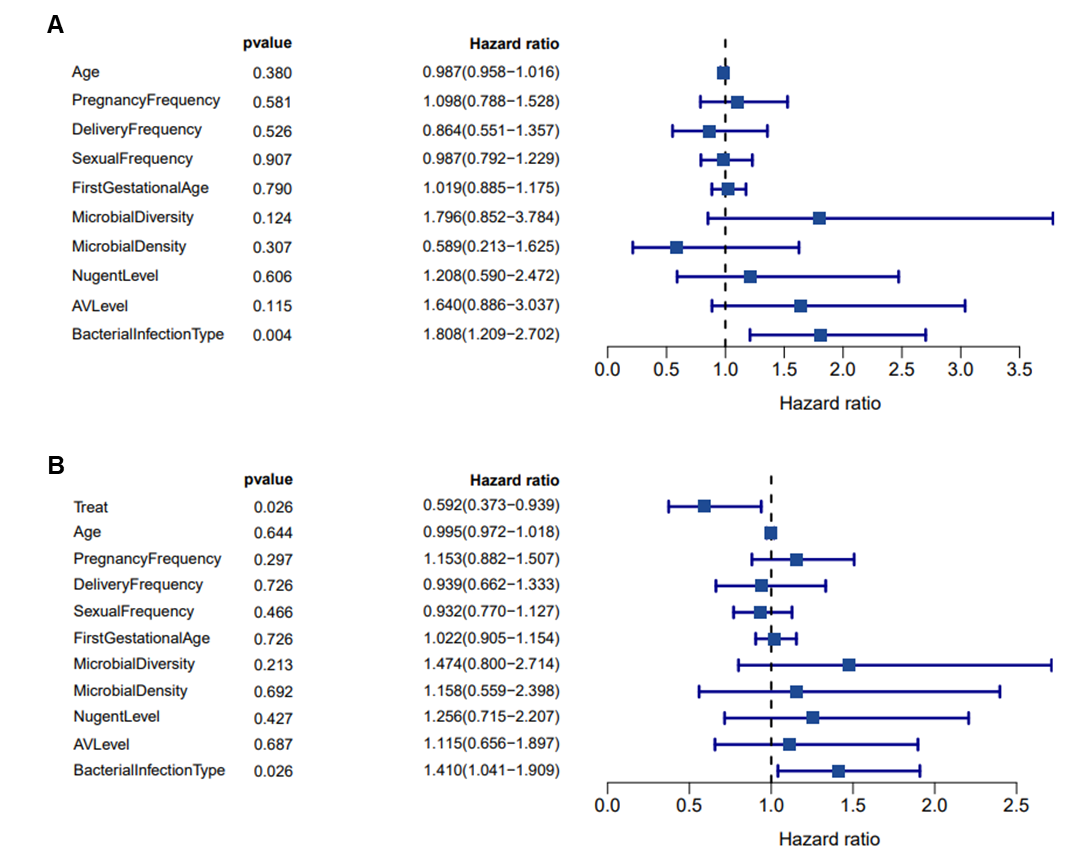


**Supplementary.Figure.1.** (A) Univariate Cox analysis of a cohort of 104 HPV patients who were not given HPV therapeutic drug interventions (control group); (B) Univariate Cox analysis on a cohort consisting of control group and 101 HPV patients who were administered Baofukang suppositories (Baofukang group).


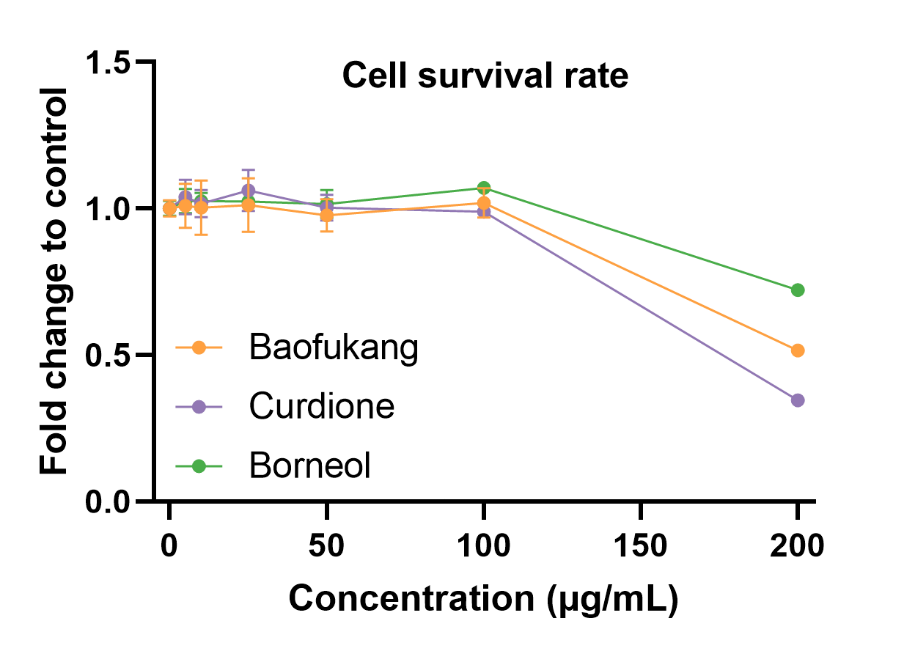


**Supplementary.Figure.2.** Biocompatibility of Baofukang suppositories, Curdione and Borneol on primary vaginal cells. All three exhibited cytotoxicity after 100 μg/ml.


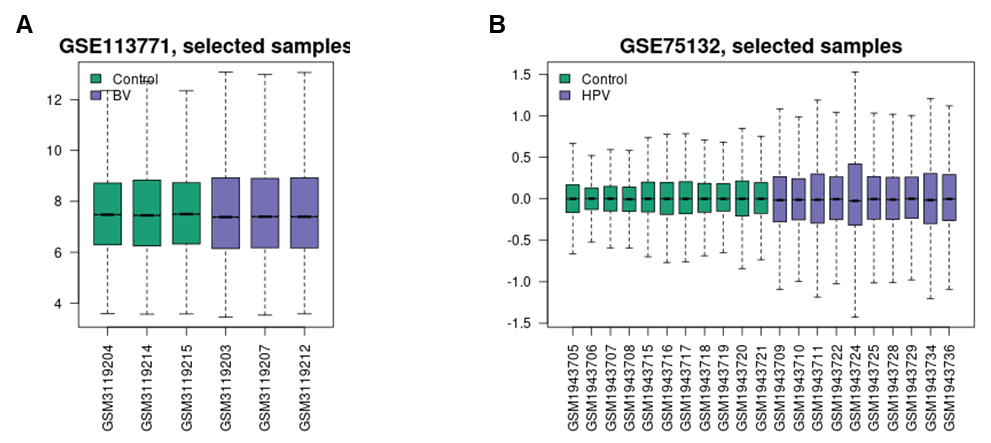


**Supplementary.Figure.3.** Normalization of selected data in the GEO databases (A) GSE113771 and (B) GSE75132.


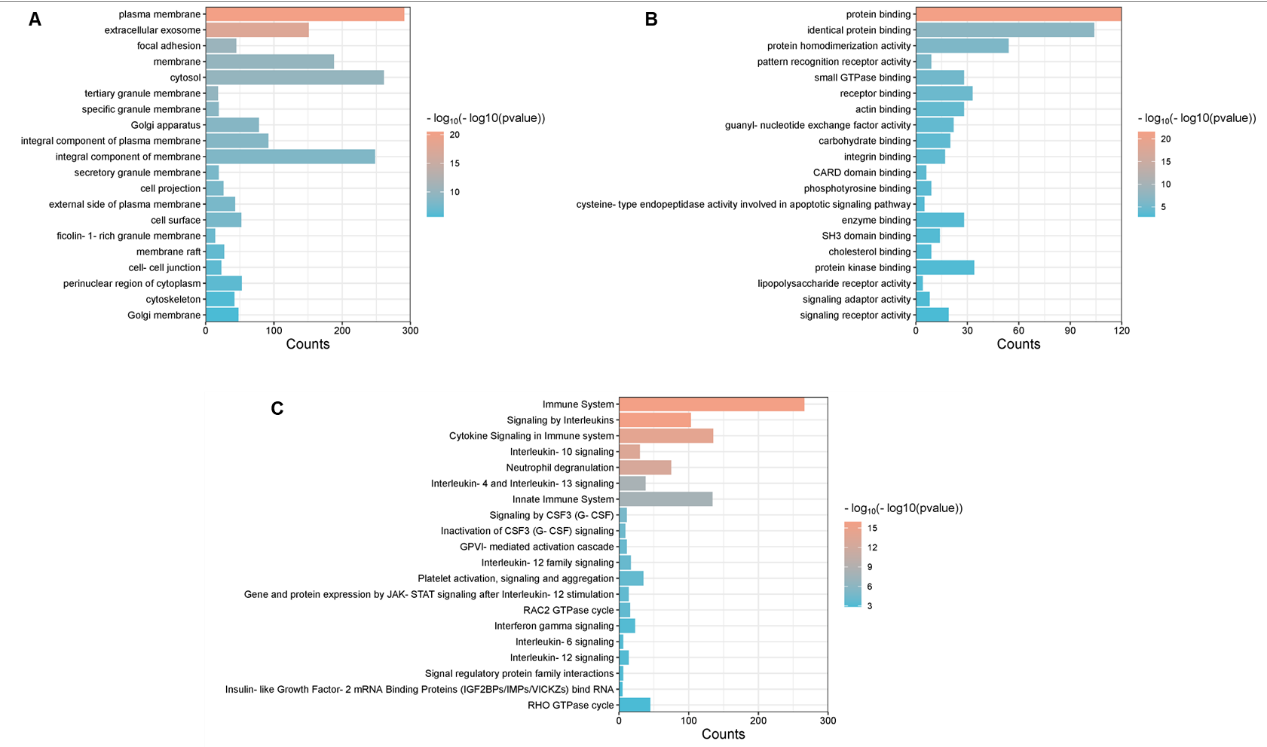


**Supplementary.Figure.4.** Enrichment analysis of (A) GO cellular component, (B) GO molecular function and (C) Reactome Signaling Pathways based on the co-DEGs.


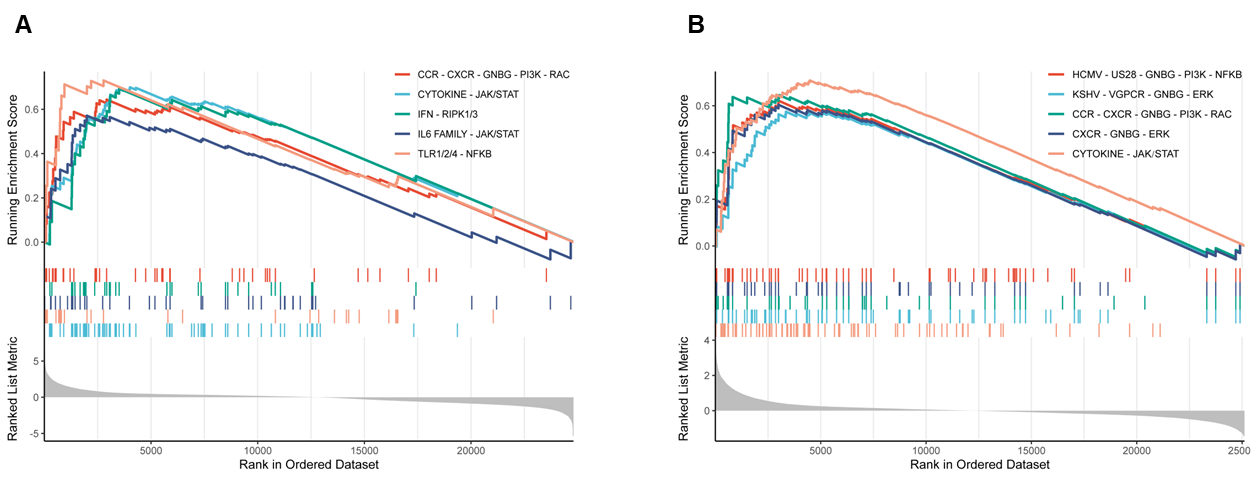


**Supplementary.Figure.5.** Top five signaling pathways significant for GSEA enrichment analysis of (A) GSE113771 and (B) GSE75132


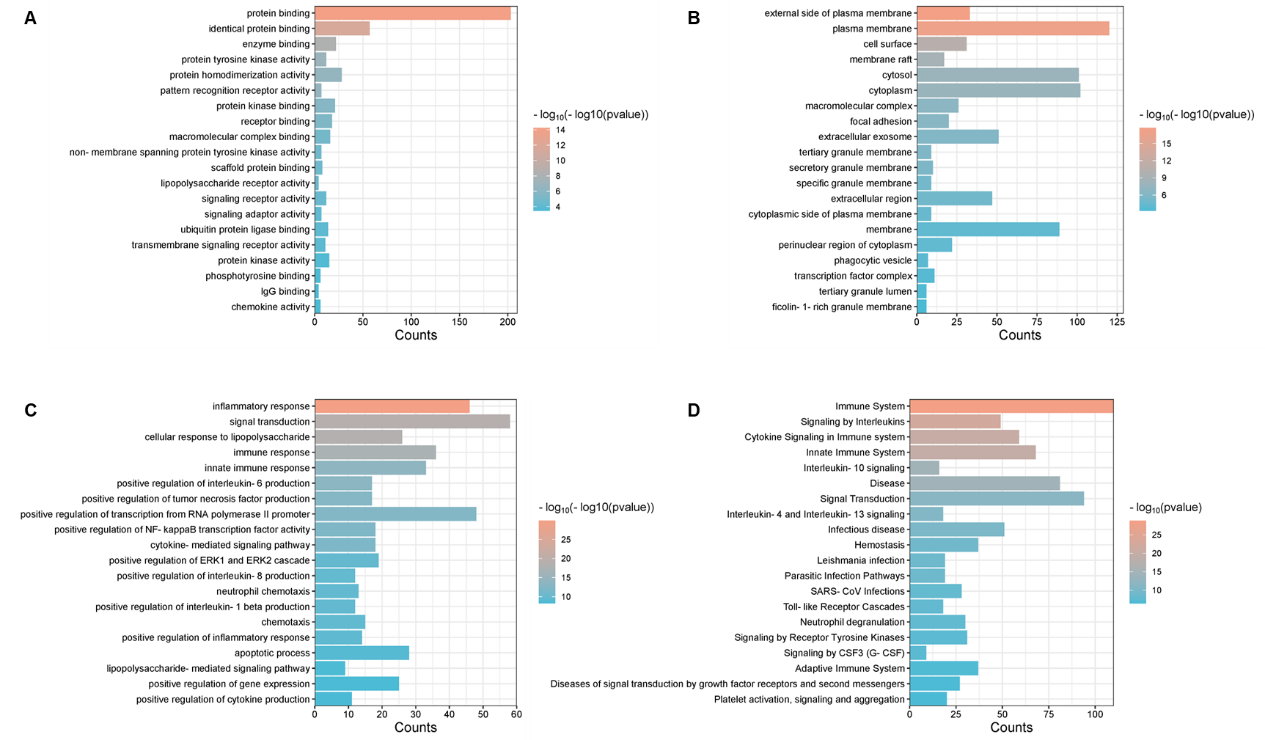


**Supplementary.Figure.6.** Enrichment analysis of (A) GO cellular component, (B) GO molecular function, (C) GO biological processes and (D) Reactome Signaling Pathways based on the co-targets and sub-targets.


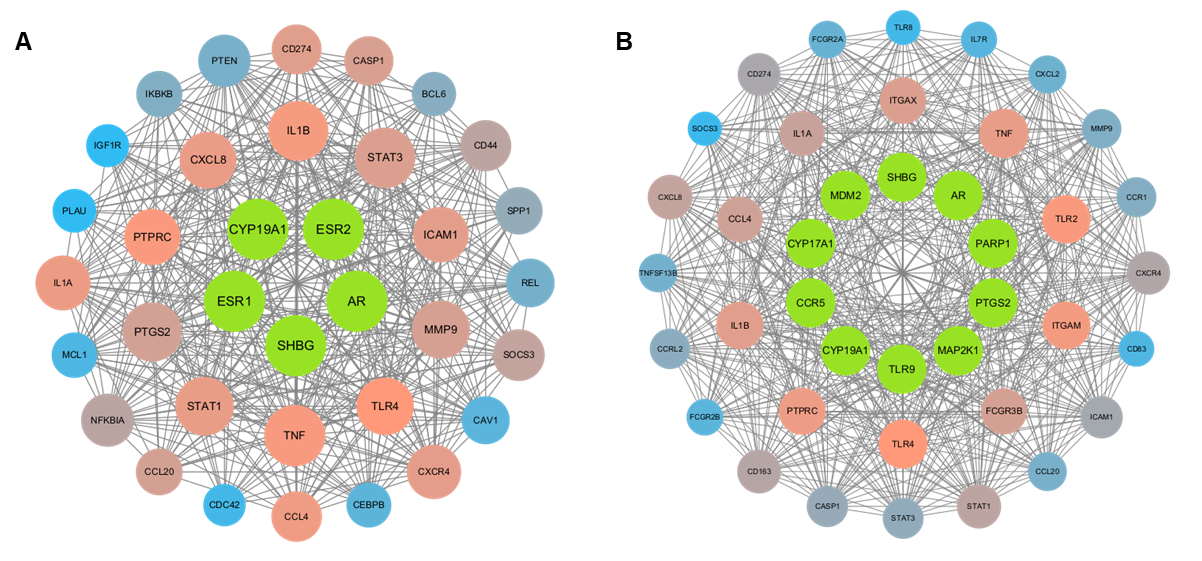


**Supplementary.Figure.7.** PPI analysis of (A) borneol co-targets and (B) curdione co-targets, each with the top 30 sub-targets with high MCC scores.


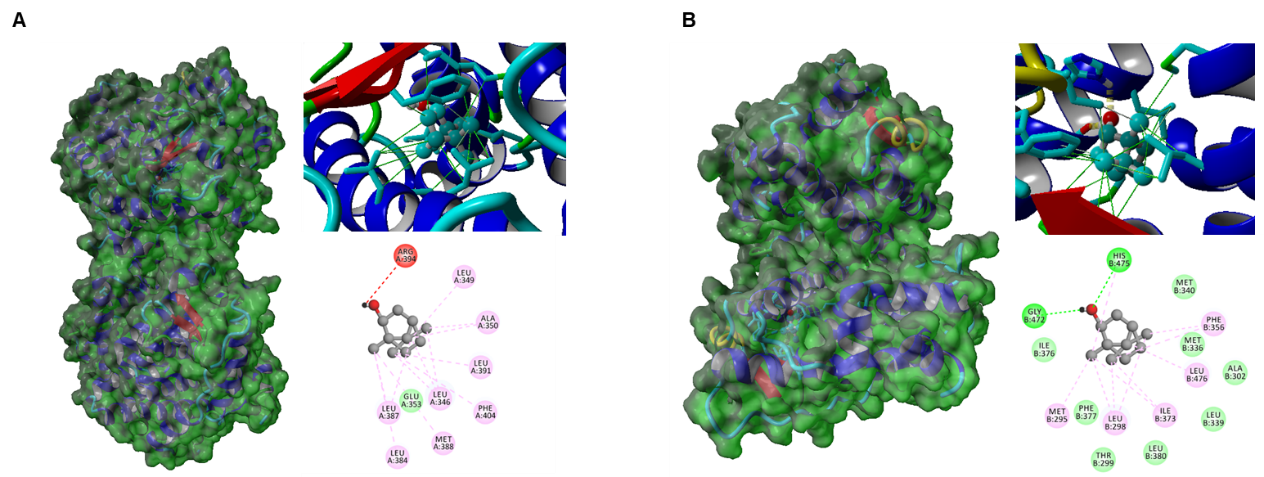


**Supplementary.Figure.8.** Molecular docking of borneol with (A) ESR1 (bind energy = -6.19 kcal/mol) and (B) ESR2 (bind energy = -6.17 kcal/mol). Molecular docking and analysis by YASARA (2). Borneol 3D Conformer with 2D Structure derived from Pubchem (https://pubchem.ncbi.nlm.nih.gov/). ESR1 (Homo sapiens) and ESR2 (Homo sapiens) 3D Conformer derived from NCBI (https://www.ncbi.nlm.nih.gov/structure/).


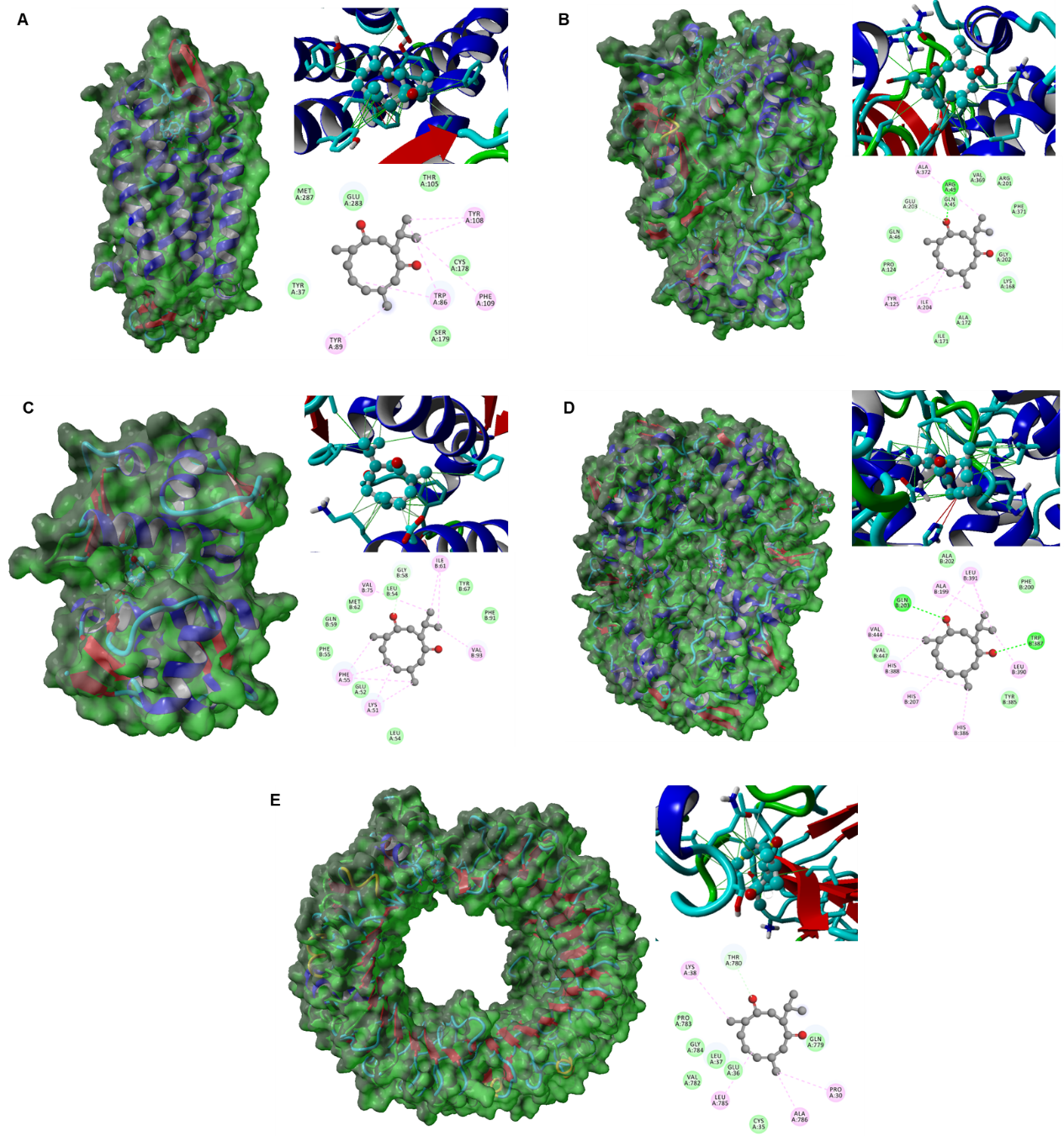


**Supplementary.Figure.9.** Molecular docking of curdione with (A) CCR5 (bind energy = -7.45 kcal/mol), (B) MAP2K1 (bind energy = -7.09 kcal/mol), (C)MDM2 (bind energy = -7.59 kcal/mol), (D)PTGS2 (bind energy = -7.13 kcal/mol) and (E)TLR9 (bind energy = -6.71 kcal/mol). Molecular docking and analysis by YASARA (2). Curdione's 3D Conformer with 2D Structure derived from Pubchem (https://pubchem.ncbi.nlm.nih.gov/). CCR5 (Homo sapiens), MAP2K1 (Homo sapiens), MDM2 (Homo sapiens), PTGS2 (Homo sapiens) and TLR9 (Mus musculus) 3D Conformer derived from NCBI (https://www.ncbi.nlm.nih.gov/structure/).


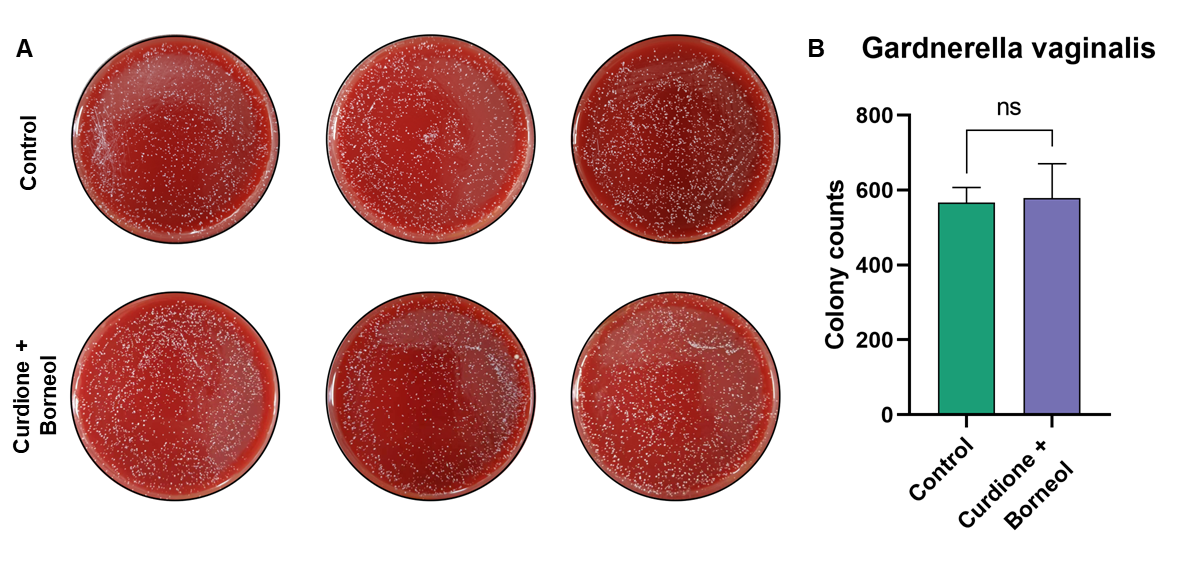


**Supplementary.Figure.10.** (A) Bactericidal properties of curdione + borneol against Gardnerella vaginalis at no-cytotoxic concentration (100 μg/ml) and (B) statistical graph of colonization.


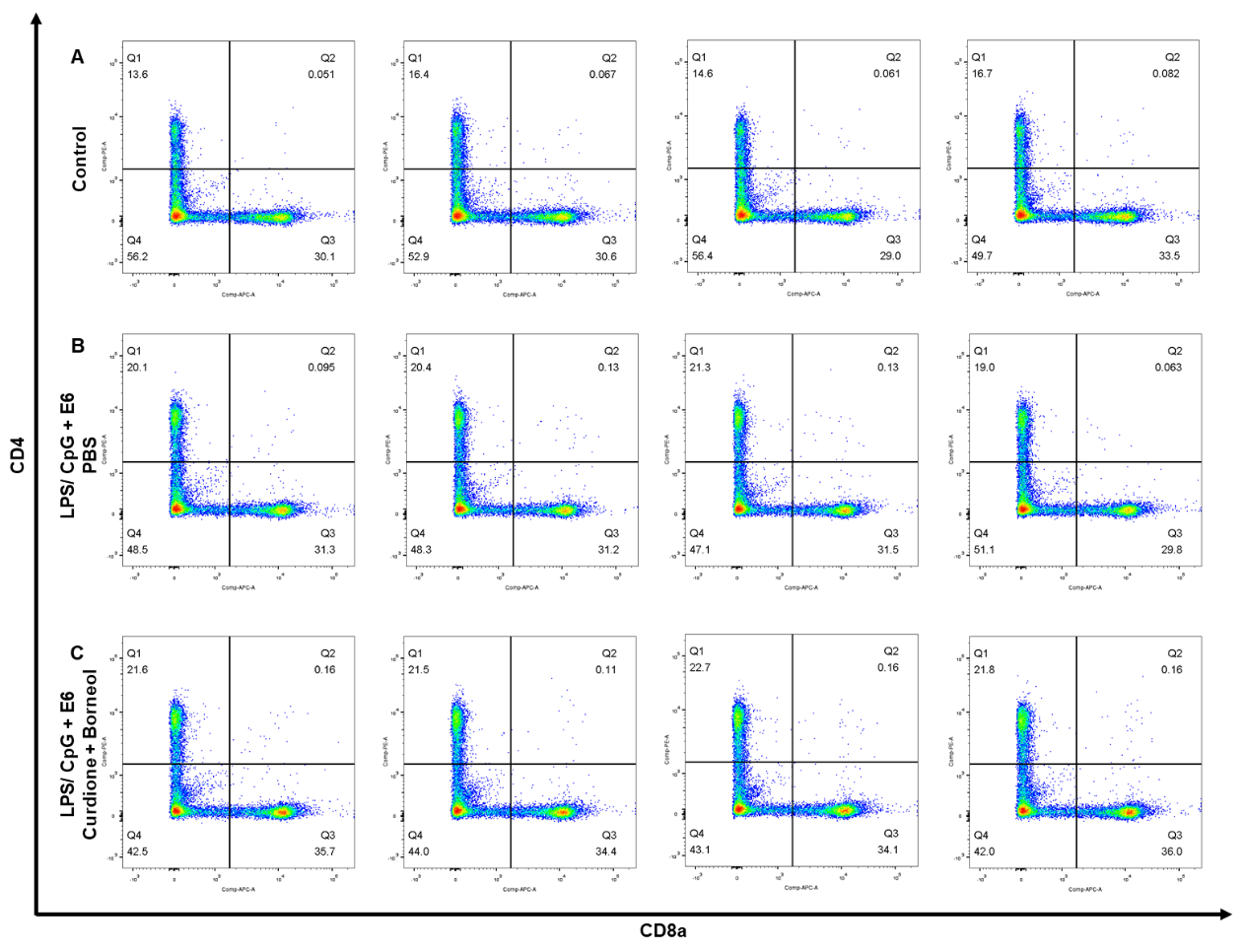


**Supplementary.Figure.11.** In the LPS/CpG + E6 co-stimulation co-culture model, the ratio of CD4^+^ to CD8^+^ T cell differentiation in splenic lymphocytes after different stimulations by flow cytometry.


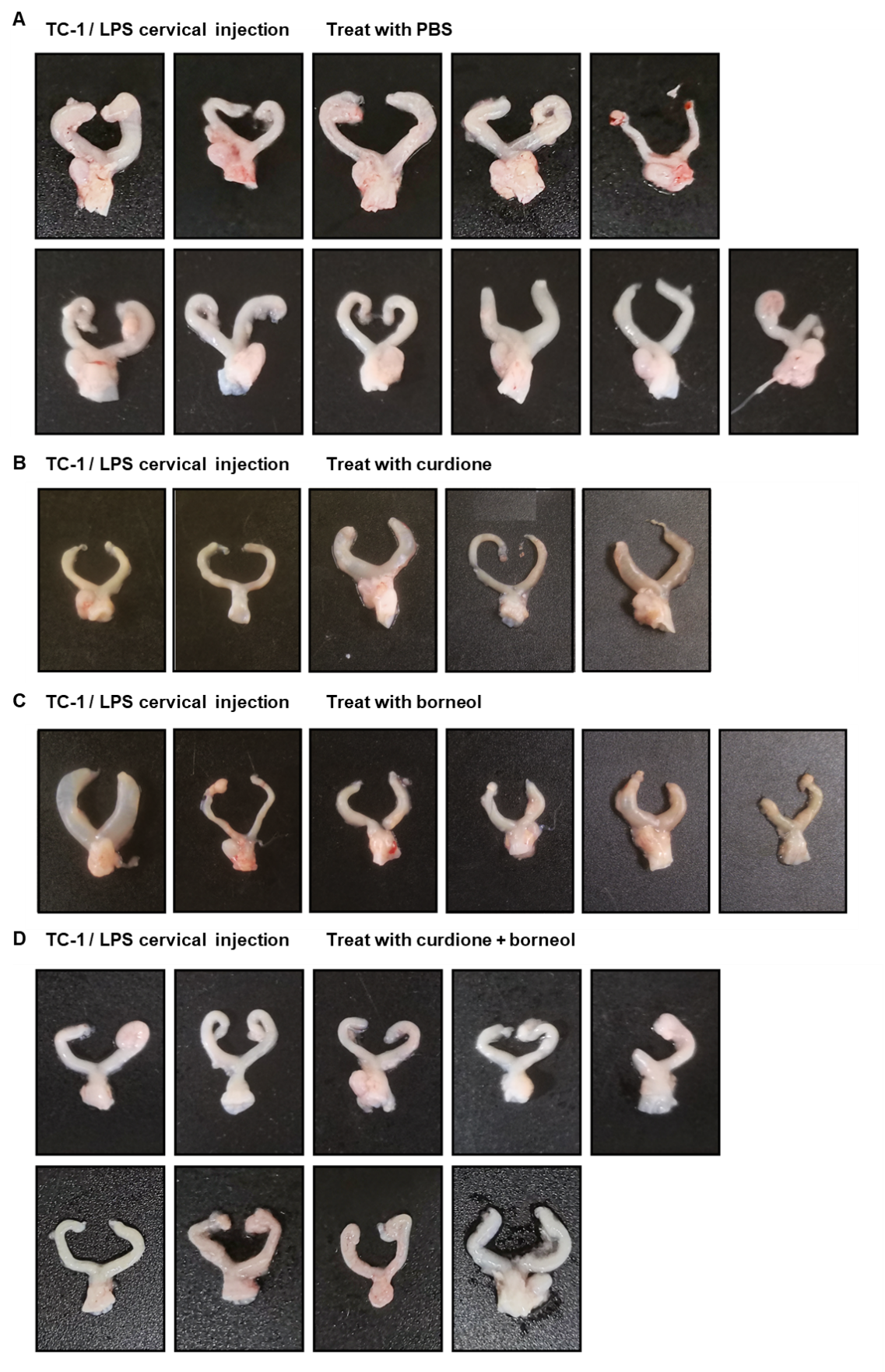


**Supplementary.Figure.12.** Image of a mouse uterus, taken after 14 days of treatmentt with (A)PBS, (B)curdione, (C)borneol or (D)curdione + borneol via vaginal lavage, in the TC-1/LPS cervical in situ injection model.


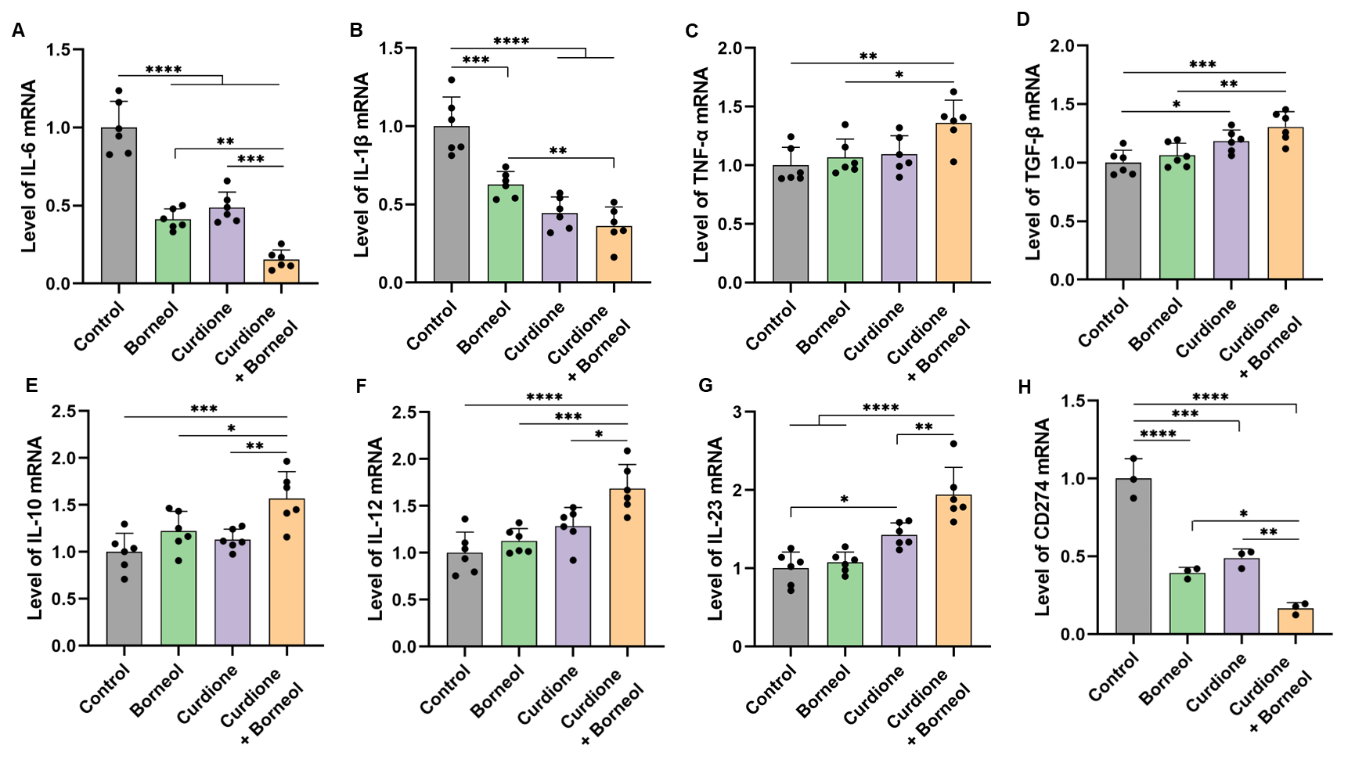


**Supplementary.Figure.13.** mRNA expression levels of (A) IL-6, (B) TNF-α, (C) IL-12, (D) IL-10, (E) IL-1β, (F) IL-23, (G) TGF-β and (H) CD274 in cervical and vaginal tissues, measured after 14 days of treatmentt in the TC-1/LPS cervical in situ injection model, *n* = 6.


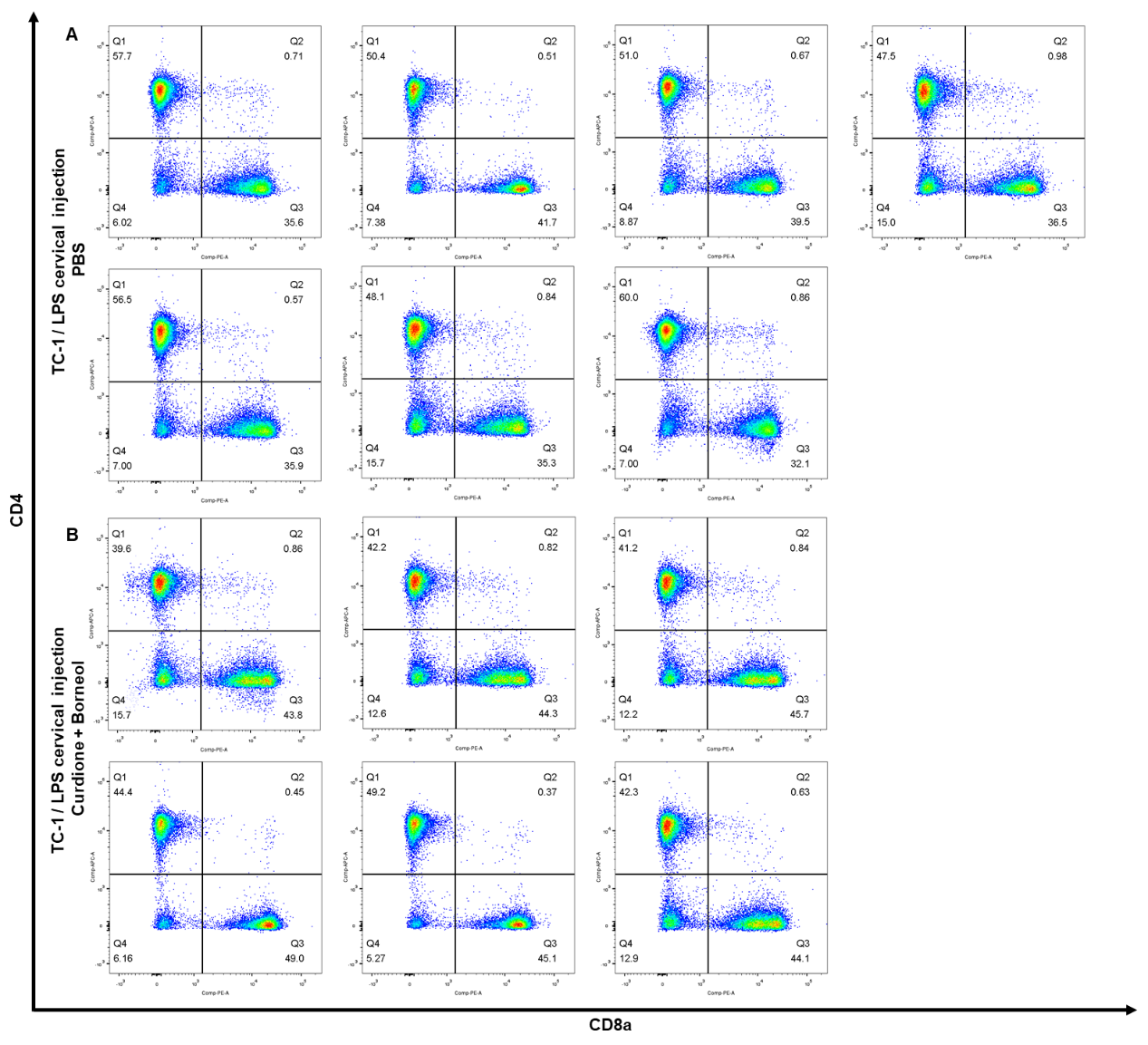


**Supplementary.Figure.14.** In the TC-1/LPS cervical in situ injection model after 14 d of (A)PBS or (B)curdione + borneol vaginal lavage treatment, the ratio of CD4+ to CD8+ T cell differentiation in the spleen, *n* = 6 or 7.


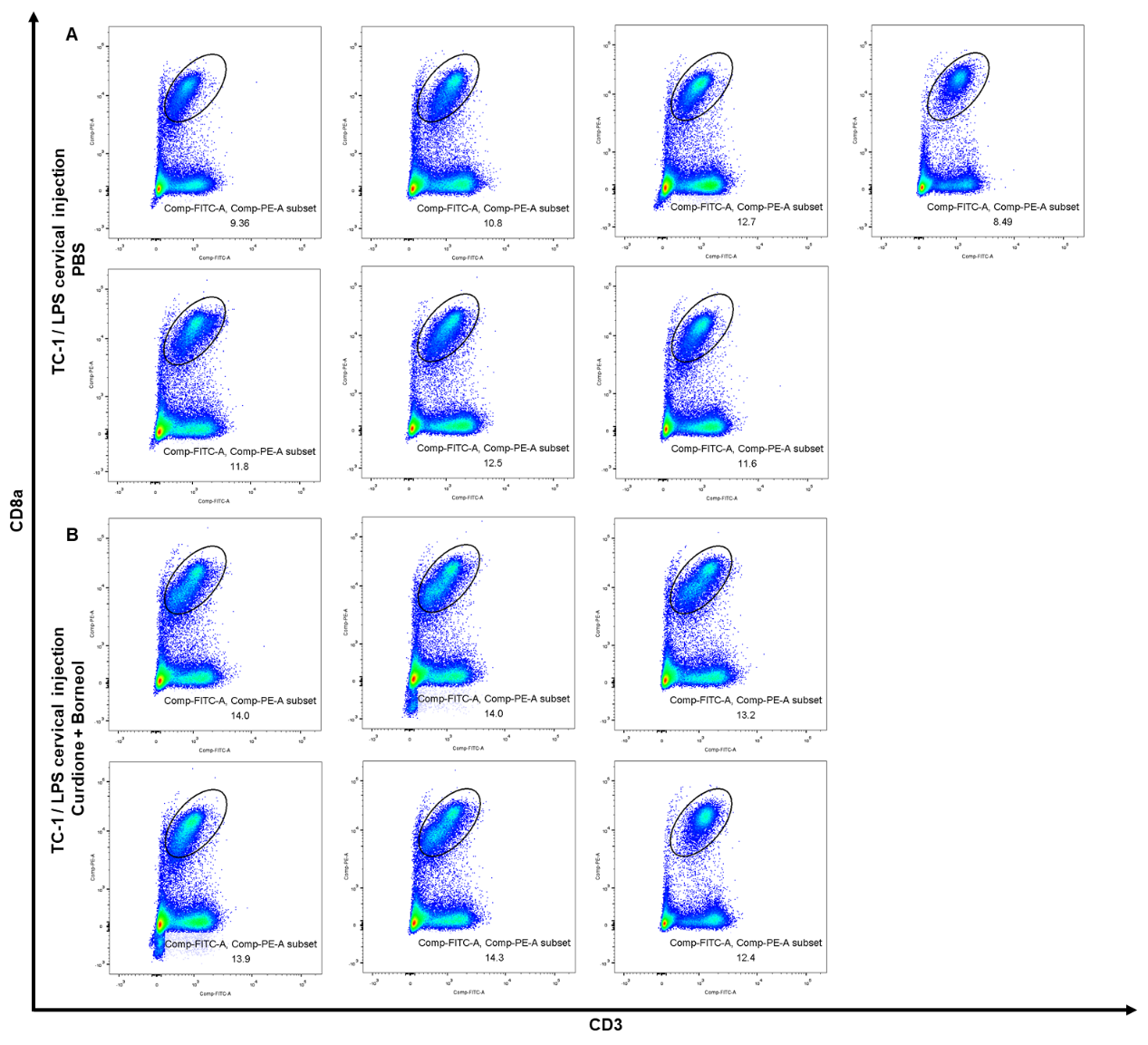


**Supplementary.Figure.15.** In the TC-1/LPS cervical in situ injection model after 14 d of (A)PBS or (B)curdione + borneol vaginal lavage treatment, the ratio of CD3^+^ CD8^+^ T cells in the spleen, *n* = 6 or 7.


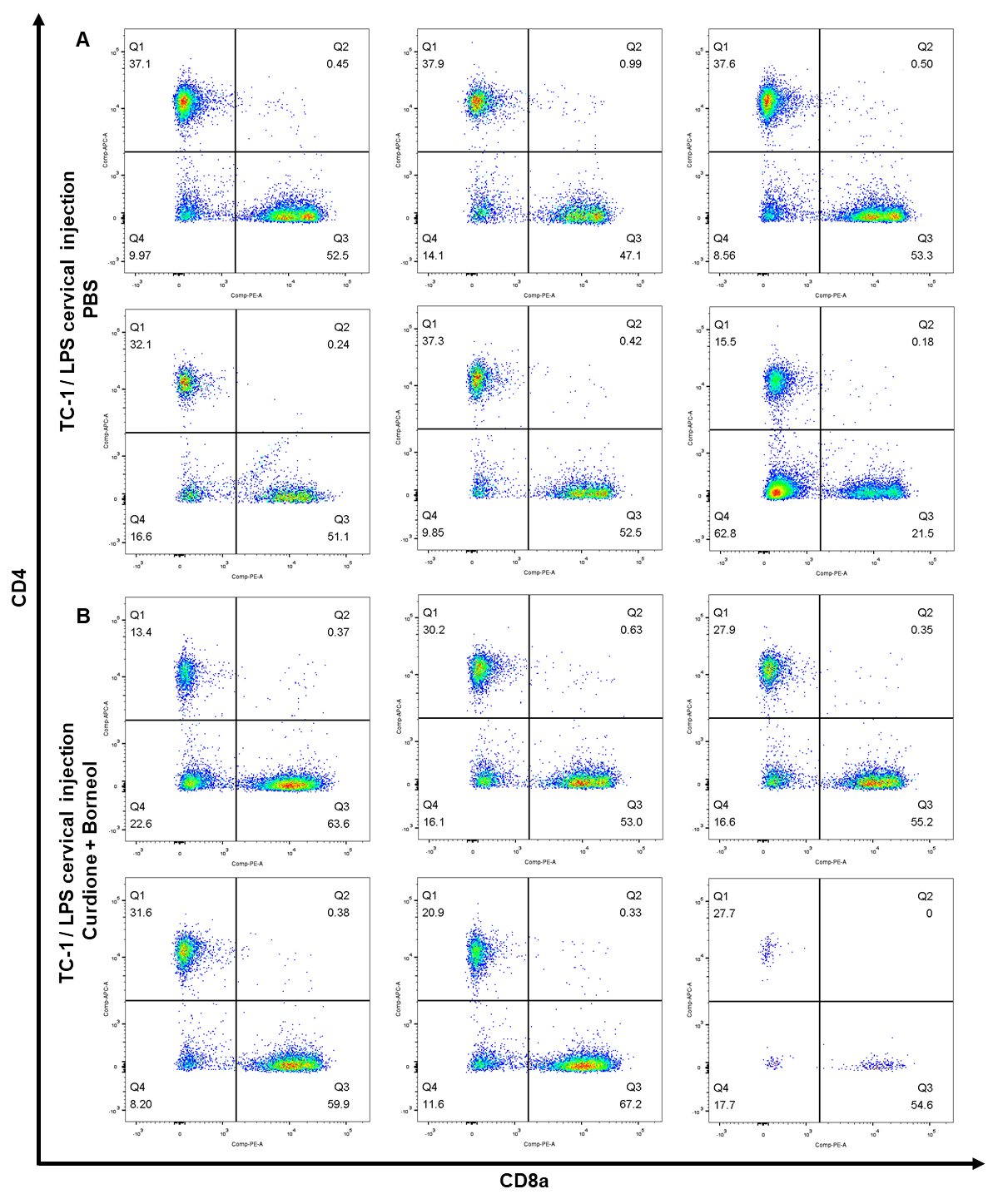


**Supplementary.Figure.16.** In the TC-1/LPS cervical in situ injection model after 14 d of (A)PBS or (B)curdione + borneol vaginal lavage treatment, the ratio of CD4^+^ to CD8^+^ infiltrated T cell differentiation in the cervix and vagina, *n* = 6.


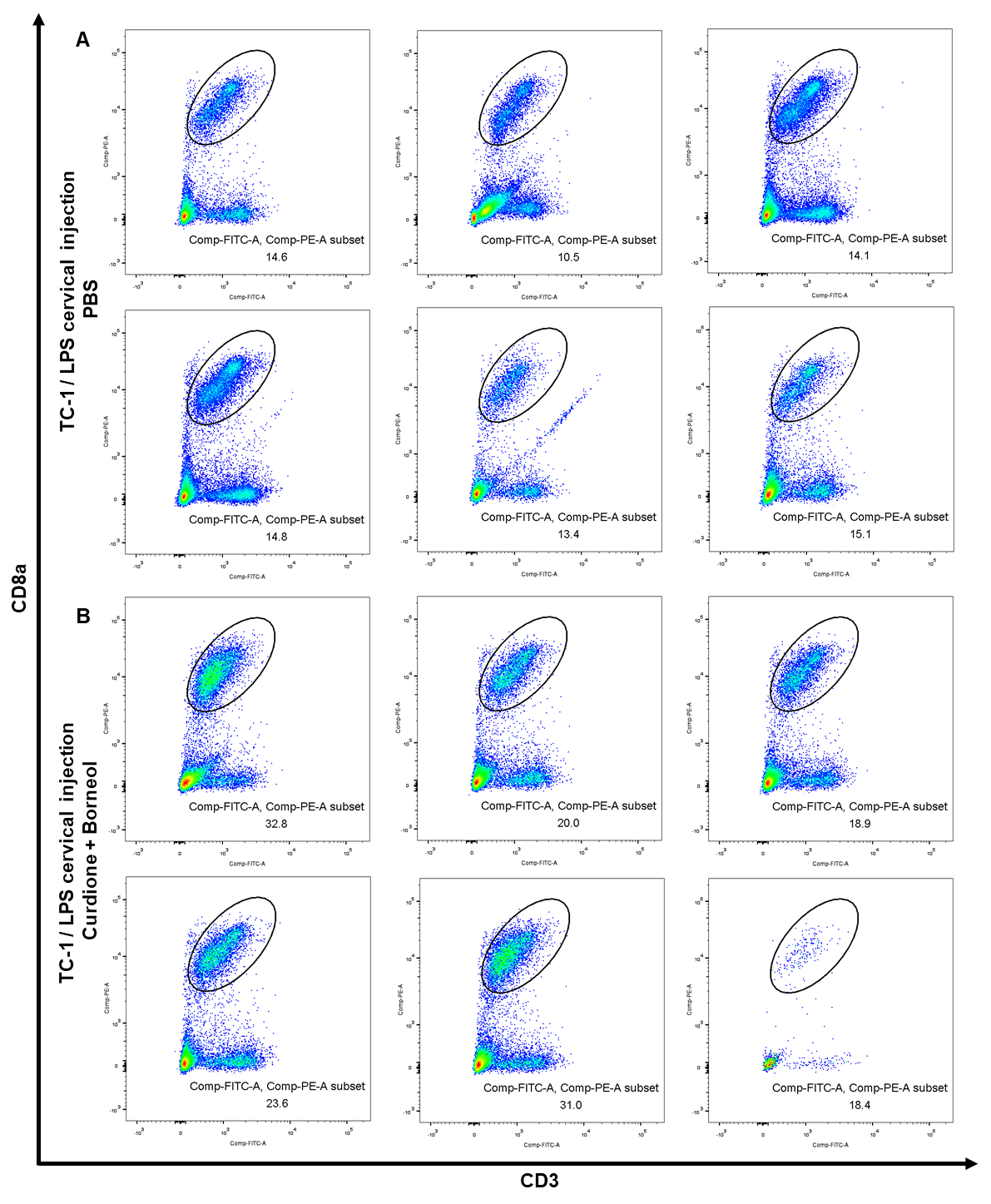


**Supplementary.Figure.17.** In the TC-1/LPS cervical in situ injection model after 14 d of (A)PBS or (B)curdione + borneol vaginal lavage treatment, the ratio of CD3^+^ CD8^+^ infiltrating T cells in the cervix and vagina, *n* = 6.


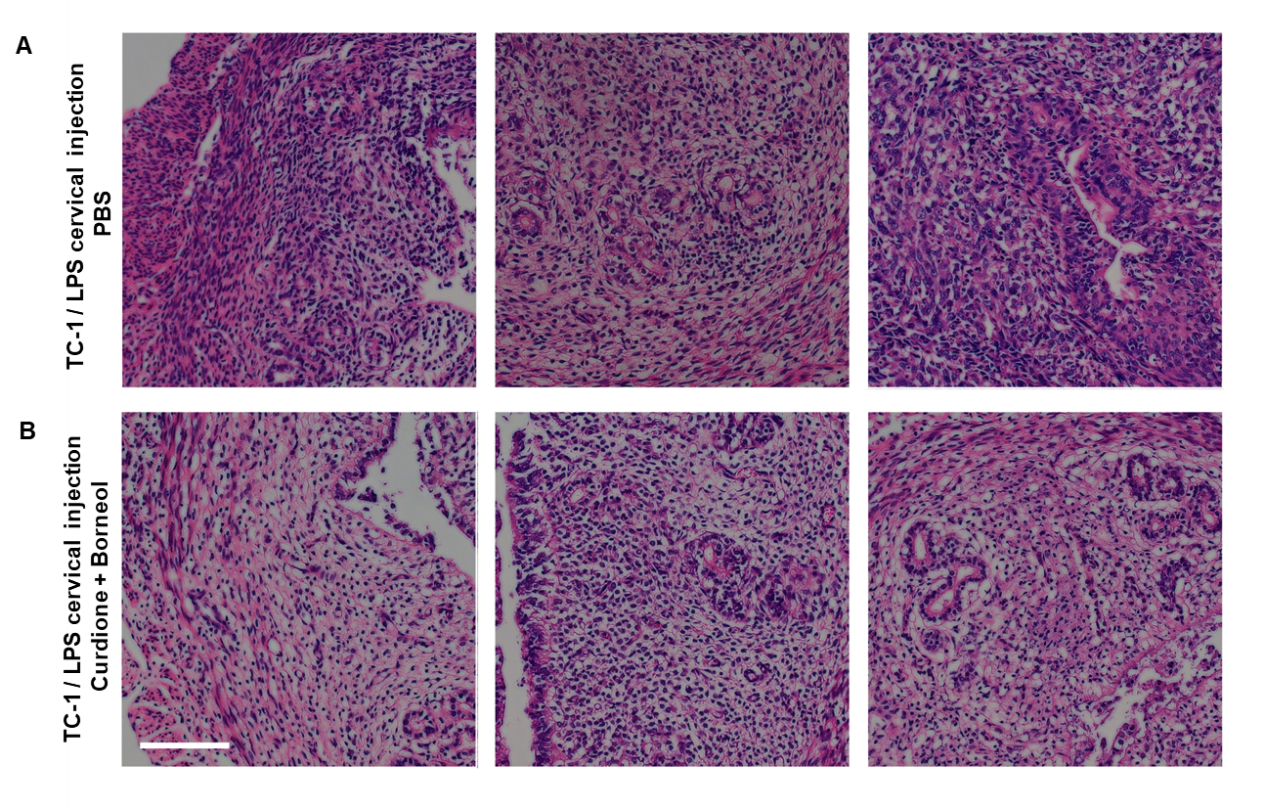


**Supplementary.Figure.18.** The H&E staining in cervical tissue sections from the TC-1/LPS cervical in situ injection model after 14 d of (A) PBS or (B) curdione + borneol vaginal lavage treatment (scale bar: 100 μm), *n* = 3.


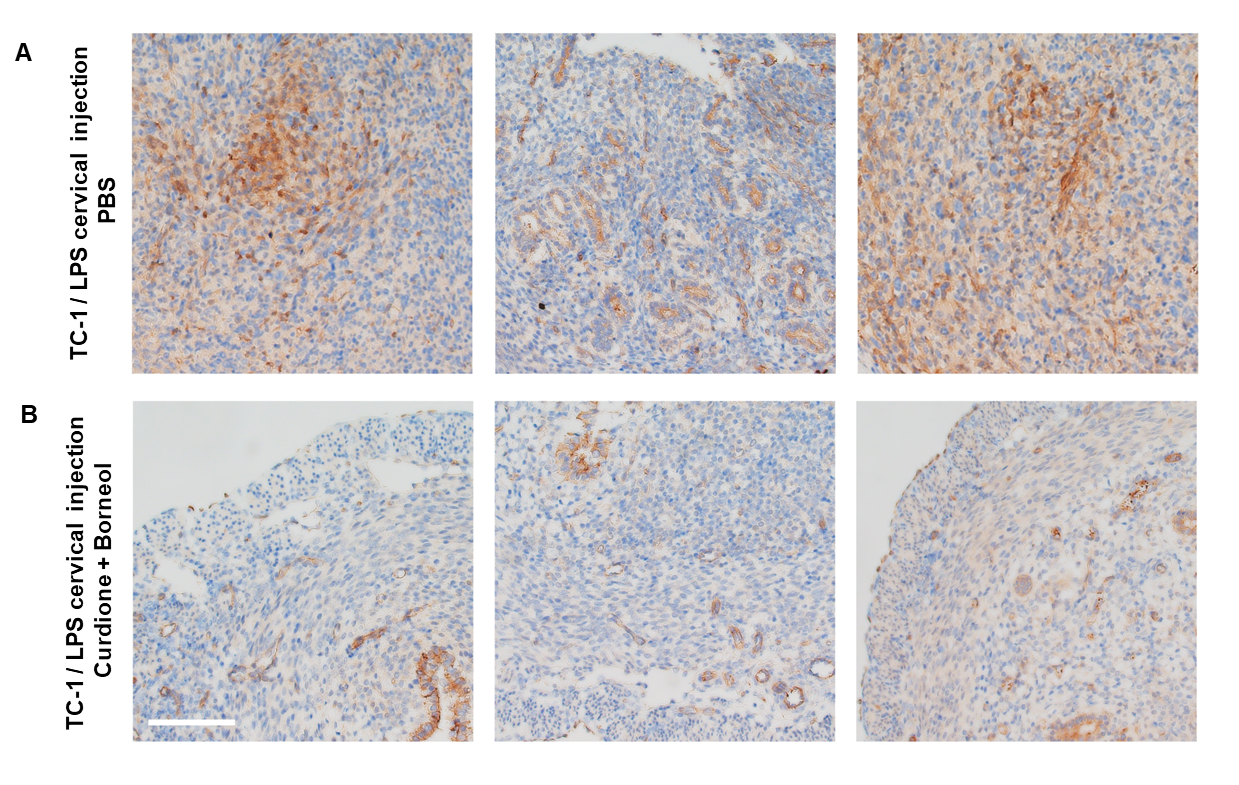


**Supplementary.Figure.19.** Immunohistochemical staining for IL-6 in cervical tissue sections from the TC-1/LPS cervical in situ injection model after 14 d of (A) PBS or (B) curdione + borneol vaginal lavage treatment (scale bar: 100 μm), *n* = 3.


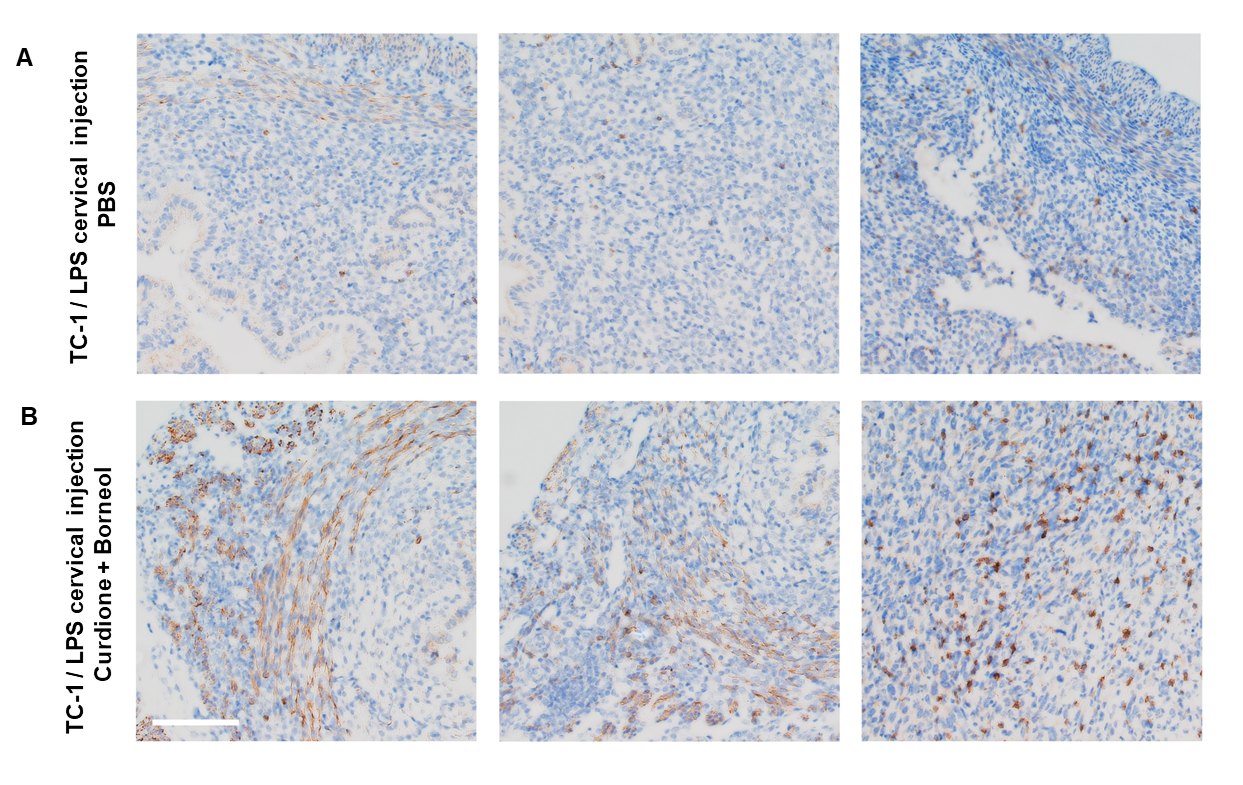


**Supplementary.Figure.20.** Immunohistochemical staining for CD8a in cervical tissue sections from the TC-1/LPS cervical in situ injection model after 14 d of (A) PBS or (B) curdione + borneol vaginal lavage treatment (scale bar: 100 μm), *n* = 3.


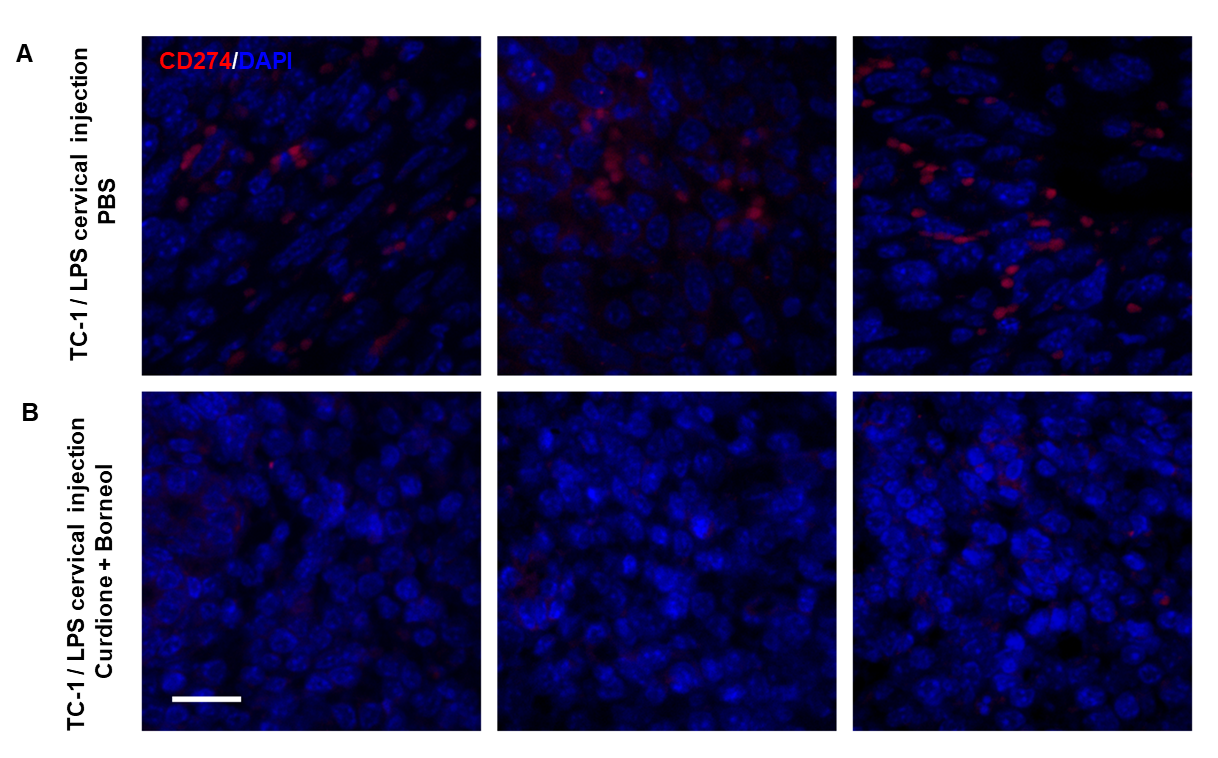


**Supplementary.Figure.21.** Immunofluorescence staining for CD274 in cervical tissue sections from the TC-1/LPS cervical in situ injection model after 14 d of (A) PBS or (B) curdione + borneol vaginal lavage treatment (red: CD274, bule: DAPI, scale bar: 20 μm), *n* = 3.

# References

1. Wan B, Qin L, Ma W, Wang H. Construction and Immune Effect of an Hpv16/18/58 Trivalent Therapeutic Adenovirus Vector Vaccine. *Infect Agent Cancer* (2022) 17(1):5. doi: 10.1186/s13027-022-00417-3.

2. Krieger E, Vriend G. Yasara View - Molecular Graphics for All Devices - from Smartphones to Workstations. *Bioinformatics* (2014) 30(20):2981-2. doi: 10.1093/bioinformatics/btu426.
